# Breast Cancer Stem Cell–Derived Tumors Escape from γδ T-cell Immunosurveillance *In Vivo* by Modulating γδ T-cell Ligands

**DOI:** 10.1158/2326-6066.CIR-22-0296

**Published:** 2023-05-04

**Authors:** Katrin Raute, Juliane Strietz, Maria Alejandra Parigiani, Geoffroy Andrieux, Oliver S. Thomas, Klaus M. Kistner, Marina Zintchenko, Peter Aichele, Maike Hofmann, Houjiang Zhou, Wilfried Weber, Melanie Boerries, Mahima Swamy, Jochen Maurer, Susana Minguet

**Affiliations:** 1 Faculty of Biology, University of Freiburg, Freiburg, Germany.; 2 Signalling Research Centres BIOSS and CIBSS, University of Freiburg, Freiburg, Germany.; 3 Center of Chronic Immunodeficiency (CCI) and Institute for Immunodeficiency, University Clinics and Medical Faculty, Freiburg, Germany.; 4 Spemann Graduate School of Biology and Medicine (SGBM), University of Freiburg, Freiburg, Germany.; 5 Institute of Medical Bioinformatics and Systems Medicine, Medical Center - University of Freiburg, Faculty of Medicine, University of Freiburg, Freiburg, Germany.; 6 Department of Medicine II (Gastroenterology, Hepatology, Endocrinology and Infectious Diseases), Freiburg University Medical Center, Faculty of Medicine, University of Freiburg, Freiburg, Germany.; 7 Medical Research Council Protein Phosphorylation and Ubiquitylation Unit, University of Dundee, Dundee, United Kingdom.; 8 German Cancer Consortium (DKTK) Partner Site Freiburg, German Cancer Research Center (DKFZ), Heidelberg, Germany.; 9 Department of Gynecology and Obstetrics, University Hospital RWTH Aachen, Aachen, Germany.

## Abstract

There are no targeted therapies for patients with triple-negative breast cancer (TNBC). TNBC is enriched in breast cancer stem cells (BCSC), which play a key role in metastasis, chemoresistance, relapse, and mortality. γδ T cells hold great potential in immunotherapy against cancer and might provide an approach to therapeutically target TNBC. γδ T cells are commonly observed to infiltrate solid tumors and have an extensive repertoire of tumor-sensing mechanisms, recognizing stress-induced molecules and phosphoantigens (pAgs) on transformed cells. Herein, we show that patient-derived triple-negative BCSCs are efficiently recognized and killed by *ex vivo* expanded γδ T cells from healthy donors. Orthotopically xenografted BCSCs, however, were refractory to γδ T-cell immunotherapy. We unraveled concerted differentiation and immune escape mechanisms: xenografted BCSCs lost stemness, expression of γδ T-cell ligands, adhesion molecules, and pAgs, thereby evading immune recognition by γδ T cells. Indeed, neither promigratory engineered γδ T cells, nor anti–PD-1 checkpoint blockade, significantly prolonged overall survival of tumor-bearing mice. BCSC immune escape was independent of the immune pressure exerted by the γδ T cells and could be pharmacologically reverted by zoledronate or IFNα treatment. These results pave the way for novel combinatorial immunotherapies for TNBC.

## Teaser

Patient-derived triple-negative BCSCs are shown to be efficiently recognized and killed by γδ T cells, but xenografted BCSCs evade immune recognition by γδ T cells by various mechanisms, paving the way for novel combinatorial immunotherapies for TNBC.

## Introduction

In breast cancer, which was the most commonly diagnosed cancer worldwide in 2020, breast cancer stem cells (BCSC) play a key role in metastasis formation, tumor recurrence, and mortality of patients (1, 2). Specifically targeting BCSCs is a promising avenue for cancer therapy, yet it faces multiple challenges mainly due to BCSC-intrinsic cell heterogeneity and drug resistance (3). BCSC-focused therapies are of even greater importance in the fight against triple-negative breast cancer (TNBC), the most aggressive and lethal breast cancer subtype, for which no targeted treatment options are currently available (4).

Immunotherapies involving the transfer of autologous *ex vivo* engineered αβ T cells back into patients have recently been employed for treating a variety of cancers. However, the efficacy of these treatments relies on the presence of tumor-specific antigens presented on MHC I molecules. The loss of MHC I expression and the concomitant lack of peptide presentation by cancer cells can undermine conventional αβ T-cell target recognition, and thus lead to tumor immune escape, which has been widely observed in cancers, including TNBC (5). To overcome this limitation, novel immunotherapies using unconventional non-MHC–restricted lymphocytes such as γδ T cells are currently being investigated. γδ T cells react to stress-induced proteins or phosphoantigens (pAgs) that accumulate in tumor cells due to their deregulated metabolism (6, 7).

Two subpopulations of γδ T cells are currently in focus for therapeutic applications: Vγ9Vδ2 T-cell receptor (TCR)–expressing γδ T cells (Vδ2^+^ T cells) and Vδ1^+^ γδ T cells (Vδ1^+^ T cells). Vδ2^+^ T cells represent the predominant γδ T-cell subset in the human blood and specifically recognize pAgs (8, 9). In contrast, Vδ1^+^ T cells represent a minor population in peripheral human blood and mainly locate to epithelial tissues. Vδ1^+^ T cells react to several lipid and protein antigens (10). Both of these γδ T-cell subsets have been described to kill a variety of cancer cell lines upon activation of their specific TCRs, innate receptors like NKG2D, or by engaging the “death receptors” Fas or TRAIL on tumor cells (11–13). We investigated here the potential of these two γδ T-cell subsets to target BCSCs.

Reactivity of γδ T cells against cancer stem cells of several cancer entities has been described previously (14, 15). Only two recent studies addressed the reactivity of γδ T cells against BCSCs (16, 17). While Chen and colleagues did not observe a difference in γδ T cell-mediated cytotoxicity between BCSCs and their non–stem cell counterparts, Dutta and colleagues reported that BCSCs were less susceptible to being killed by γδ T cells. To reconcile these discrepancies and to shed light onto the potential of γδ T cells to target BCSCs, studies better reflecting the clinical reality are urgently needed.

The potential success of γδ T-cell immunotherapy against solid tumors also relies on the efficient localization of these cells into the tumor tissue because T cells require direct cell-cell contact to exert their cytotoxicity. γδ T cells need to extravasate from the blood stream into the tissue and migrate in the stromal tumor compartment. It has been shown for conventional αβ T cells that the extracellular matrix (ECM), the major noncellular fraction of the tumor microenvironment, negatively affects the migration and infiltration of αβ T cells in non-small cell lung cancer and ovarian cancer (18, 19). High stromal content, as well as low numbers of infiltrating T cells, also have been associated with poor clinical outcome in breast cancer (20). Therefore, increasing the infiltration of T cells into tumors is a major goal in the development of immunotherapies. In line with this, exogenous expression of the ECM-modifying enzyme heparanase can increase the tumor infiltration of chimeric-antigen receptor (CAR)–expressing αβ T cells, promoting tumor rejection in melanoma and neuroblastoma xenograft models (21). Whether the ECM-rich stromal compartment of tumors also hampers γδ T-cell migration, infiltration, and tumor rejection has not yet been investigated.

We sought here to use a model aiming to reflect the situation in human patients with TNBC to test the effect of γδ T cell–based therapy.

## Material and Methods

### Cells

Healthy human dermal fibroblasts of neonatal origin (HDFn, from Life Technologies, 2018) were cultured in DMEM GlutaMAX medium (Thermo Fisher Scientific, 61965-026) supplemented with 10% FBS (Thermo Fisher Scientific, 10270-106), 100 μg/mL penicillin, 100 μg/mL streptomycin (Thermo Fisher Scientific, 15140-122), and 10 mmol/L HEPES (Thermo Fisher Scientific, 15630-080). HEK293T cells (from ATCC, 2016) were grown in DMEM GlutaMAX medium supplemented with 10% FBS, 100 μg/mL penicillin, 100 μg/mL streptomycin, 10 mmol/L HEPES, and 10 μmol/L sodium pyruvate (Thermo Fisher Scientific, 11360-039). HDFn and HEK293T cells were cultured at 37°C in a humidified atmosphere of 7.5% CO_2_. Cells were regularly tested for *Mycoplasma* but were not reauthenticated.

BCSC generation has been described previously (22). Briefly, all BCSC lines originated from independent breast tumor samples lacking estrogen receptor, progesterone receptor, and HER2 proteins (22). All experiments were performed in accordance with the Declaration of Helsinki. All experimental protocols were approved by the Institutional Review Board in the Ethics vote 307/13 (independent Ethics Committee University of Freiburg, Freiburg, Germany). Written informed consent was obtained from each patient. Cells were isolated by mechanical dissociation of the tumor material and mixed 1:1 with Matrigel (growth factor reduced, Corning, 354230) and topped up with mammary stem cell (MSC) medium (MEBM medium; Lonza, CC-3151) supplemented with 1× B27 (Thermo Fisher Scientific, 17504-044), 1× amphotericin (Sigma-Aldrich, A2942), 20 ng/mL EGF (Peprotech, AF-100-15B), 20 ng/mL FGF (Peprotech, AF-100-18B), 4 μg/mL heparin (Sigma-Aldrich, H3149), 35 μg/mL gentamicin (Thermo Fisher Scientific, 15750-045), 500 nmol/L H-1152 (Calbiochem, 555552), 100 μg/mL penicillin, and 100 μg/mL streptomycin (Thermo Fisher Scientific, 15140-122). The cells were cultured at 37°C under low oxygen conditions (3% O_2_, 5% CO_2_, 92% N_2_). Three-dimensional (3D) cells stably proliferating were cultured and expanded in two-dimensional MSC medium. Passaging of BCSCs was performed as described previously (22). Experiments were conducted in a passaging window of 15 passages. BCSCs were cultured at 37°C under low oxygen conditions (3% O_2_, 5% CO_2_, 92% N_2_).

### **γδ** T-cell expansion

Primary γδ T-cell expansion was performed as described previously (23). Briefly, peripheral blood mononuclear cells (PBMC) were purified from the blood of healthy donors via density gradient centrifugation (Pancoll human, Pan Biotech, P04-601000). Buffy coats used as blood source were purchased from the blood bank of the University Medical Centre Freiburg (approval of the University Freiburg Ethics Committee: 147/15 and 21-1010). PBMCs were resuspended at a concentration of 1 × 10^6^ cells/mL in γδ T-cell medium: RPMI1640 medium (Life Technologies, 11554516) supplemented with 10% FBS, 100 μg/mL penicillin, 100 μg/mL streptomycin, 10 mmol/L HEPES, 10 μmol/L sodium pyruvate, and 1× MEM nonessential amino acids (Pan Biotech, P08-32100). Expansion was induced with 1 μg/mL concanavalin A (Sigma-Aldrich, C5275), 10 ng/mL IL2 (Peprotech, 200-02), and 10 ng/mL IL4 (Peprotech, 200-04). Cells were adjusted every 3 to 4 days to 1 × 10^6^ cells/mL with γδ T-cell medium and cytokines. The cells were cultured at 37°C in a humidified atmosphere of 5% CO_2_. At days 10 to 14 after expansion started, γδ T cells were separated from αβ T cells via negative selection (MACS TCRγ/δ^+^ T Cell Isolation Kit, Miltenyi Biotec, 130-092-892). Cultures with a purity of ≥ 95% were used for experiments from day 14 on.

### Lentiviral constructs, generation of lentiviral particles, and transduction of **γδ** T cells

*MMP14* cDNA or the cDNA of the catalytically inactive form of MMP14 *(MMP14^E240A^*; both kind gifts from Pilar Gonzalo, CNIC, Spain and Joaquin Teixidó, CIB, Spain) were cloned into the lentiviral backbone pLVX-CMV-IRES-zsGreen1 (Takara/Clontech #632187) by Gibson assembly. The CMV promoter was exchanged with a short EF-1α promoter sequence

(GATTGGCTCCGGTGCCCGTCAGTGGGCAGAGCGCACATCGCCCACAGTCCCCGAGAAGTTGGGGGGAGGGGTCGGCAATTGAACCGGTGCCTAGAGAAGGTGGCGCGGGGTAAACTGGGAAAGTGATGTCGTGTACTGGCTCCGCCTTTTTCCCGAGGGTGGGGGAGAACCGTATATAAGTGCAGTAGTCGCCGTGAACGTTCTTTTTCGCAACGGGTTTGCCGCCAGAACACAGGTGTCGTGACGCG). The integrity of each plasmid was verified by restriction enzyme digestion and Sanger sequencing.

For the generation of lentiviral particles, 1 × 10^7^ HEK 293T cells were plated on 15 cm dishes and cultured at 37°C and 7.5% CO_2_. After 24 hours, the medium was exchanged and HEK293T cells were transfected with the indicated constructs and the packaging plasmids pCMV-dR8.74 and pMD2-vsvG (both kind gifts from Didier Trono, EPFL, Switzerland) using polyethylenimine transfection (polysciences, 24765). After 24 and 48 hours, the viral particle-containing supernatant was harvested, pooled, filtered, and concentrated using density centrifugation [10% sucrose w/v in PBS/0.5 mmol/L Ethylenediaminetetraacetic acid (EDTA)] for 4 hours at 10,000 × *g* and 6°C. The supernatant was discarded, and the viral particles were resuspended in PBS using 1/400th of the harvested volume and stored at −80°C.

γδ T cells were lentivirally transduced with a multiplicity of infection (MOI) of 2-5 as indicated for the individual experiments. Transduced γδ T cells were checked for zsGreen1 and matrix metalloprotease 14 (MMP14) expression by flow cytometry after 2 to 3 days after transduction.

### Flow cytometry analysis

To stain cell surface proteins, cells were washed once with flow cytometry buffer (PBS supplemented with 2% FBS) and incubated in the diluted antibody solution for 15 minutes at 4°C. In the case of fluorophore-labeled antibodies, cells were washed once with flow cytometry buffer and analyzed on a Gallios flow cytometer (Beckman Coulter). The flow cytometry results were analyzed using FlowJo v10.8 Software (BD Life Sciences). Unlabeled primary antibodies were visualized using fluorescently labeled secondary antibodies. After washing away the primary antibody, cells were incubated in the diluted secondary antibody solution for 15 minutes at 4°C. Finally, cells were washed once as described above and analyzed on a Gallios flow cytometer.

### Antibodies and chemicals

Self-made anti-CD3ε (clone UCHT1) and anti-CD28 (clone CD28.2) from BioLegend were used for γδ T-cell stimulation. Monoclonal anti-HMGCR (clone CL0260, Invitrogen) and anti-Vinculin (ab129002, Abcam) were used for immunoprecipitation (IP) and Western blot analysis.

For flow cytometry, the following antibodies were used: anti-EpCAM-AlexaFluor488 (clone 9C4), anti-CD107a-PE (clone H4A3), anti-BTN3A-PE (clone BT3.1), anti-Fas-BV421 (clone DX2), anti-TRAILR1-APC (clone DJR1), anti-TRAILR2-PE (clone DJR2-4), anti-ICAM-1-BV421 (clone HA58), anti-CD3ε-AlexaFluor488 (clone UCHT1), anti-CD3ε-AlexaFluor647 (clone UCHT1), anti-CD27-PE (clone M-T271), anti-NKG2D-APC (clone 1D11), anti-LAG-3-AlexaFluor647 (clone 11C3C65), anti-PD-L1-APC (clone 29E.2A3), anti-PD-L2-BV421 (clone 24F.10C12), anti-Vδ2TCR-Biotin (clone B6), anti-CXCR1-APC (clone 8F1),  anti-CXCR3-AlexaFluor647 (clone G025H7), anti-CXCR4-PE (clone 49801),  anti-CXCR5-APC (clone J252D4), anti-CXCR6-PE (clone K041E5), α-CCR2-APC/Fire 750 (clone K036C2),  anti-CCR3-FITC (clone 5E8), anti-CCR10-APC (clone 6588-5), and anti-TIM-3-PE-Cy7 (clone F38-2E2) from BioLegend.  Anti-ULPB2/5/6 (mouse, clone 165903) and  anti-CCR4-APC (clone 205410) from R&D Systems. Anti-mouse IgG-APC from Southern Biotech. Anti-γδTCR-PE (clone SA6.E9), anti-γδTCR-FITC (clone SA6.E9), anti-rabbit-DyLight633 (polyclonal, 35562), anti–PD-1-PE-Cy7 (clone J105), streptavidin-eFluor450 (48-4317-82), and streptavidin-PE-Cy7 (SA1012) from Thermo Fisher Scientific. Anti-CCR5-PE (clone 2D7),  anti-CCR6-BB515 (clone 11A9), anti-CCR7-AlexaFluor647 (clone 150503), and anti-CD45RA-V450 (clone HI100) from BD Biosciences. Anti-Vδ1TCR-APC (clone REA173) and anti-Vδ1TCR-PE (clone REA173) from Miltenyi Biotec. Anti-MMP14 (rabbit, polyclonal, ab53712) from Abcam. The Cell Proliferation Dye eFluor450 (65-0842-85) was purchased from Thermo Fisher Scientific. Anti-TCRVδ1-Biotin (clone REA173) from Miltenyi Biotec and anti-TCRVδ2-Biotin (clone B6) from BioLegend were combined with the antibody solution provided in the MACS TCRγ/δ^+^ T Cell Isolation Kit to separate Vδ2^+^ and Vδ1^+^ cells from γδ T-cell expansion cultures, respectively.

The following antibodies were used for immunofluorescence: α-EpCAM-BV421 (clone EBA-1) from BD Biosciences, α-fibronectin (rabbit, polyclonal) and α-rabbit IgG-AlexaFluor546 (polyclonal) from Sigma-Aldrich. CellTracker Green CMFDA Dye was used from Thermo Fisher Scientific (C7025).

The following antibodies were used for blocking experiments: anti-Fas (clone A16086F), anti-FasL (clone NOK-1), anti-TRAIL (clone RIK-2), anti-TRAILR1 (clone DJR1), anti-TRAILR2 (clone DJR2-4), anti-NKG2D (clone 1D11), anti-MICA/B (clone 6D4), anti-ICAM-1 (clone HCD54), anti-IgG1 isotype (clone MOPC-21), and anti-IgG2b isotype (clone MG2b-57) from BioLegend. Anti-ULPB2/5/6 (clone 165903) from R&D Systems. Anti-CD103 (clone 2G5) from Beckman Coulter. Zoledronic acid monohydrate (zoledronate, SML0223) and mevastatin (M2537) were purchased from Sigma-Aldrich.

Viability of γδ T cells was assessed using the FITC Annexin V Apoptosis Detection Kit I (BD Biosciences, 556547).

### Cytotoxicity and degranulation assay

Bioluminescence-based cytotoxicity assays were performed as described previously (24). Briefly, 1 × 10^4^ luciferase-expressing target cells were plated in a white 96-well flat-bottom plate. Effector cells were added to the target cells at the desired effector to target cell ratios. A total of 37.5 μg/mL d-Luciferin Firefly (Biosynth, FL08608) was added to the samples, which were then incubated for the indicated time at 37°C and measured using a luminometer (Tecan infinity M200 Pro). Bioluminescence was measured as relative light units (RLU). RLU signals from target cells alone served as spontaneous death controls. Maximum killing RLUs were determined using target cells lysed with 1% Triton X-100. The specific lysis was calculated with the following formula:

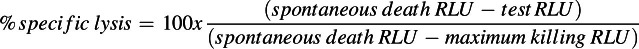


^51^Cr-release assays were performed to assess cytotoxicity in experiments including xenograft-derived tumor cells. Target cells (tumor cells) were loaded with ^51^Cr (PerkinElmer, NEZ030005MC) for 1 hour at 37°C. After washing the cells three times with medium, cells were resuspended in γδ T-cell medium and 1 × 10^4^ cells were plated in a 96-well U bottom plate. Effector cells (γδ T cells) were added to the target cells at the desired effector to target cell ratios. Samples were incubated for the indicated time at 37°C. Supernatants were transferred to a solid scintillator-coated 96-well plate (LumaPlate, Perkin Elmer) and then measured using a microplate scintillation γ-ray counter (TopCount, Perkin Elmer). Target cells without effector cells served as spontaneous ^51^Cr release controls. Maximum ^51^Cr release was determined using target cells lysed with 1:20 centrimide. The specific lysis was calculated with the following formula:

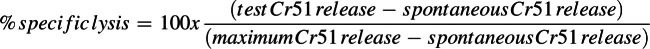


For blocking experiments, effector or targets cells were preincubated with 20 μg/mL of the indicated blocking antibodies at 37°C for 1 hour. IgG isotype antibodies were used as experimental controls. Cells were then used for cytotoxicity experiments in the presence of 10 μg/mL of the blocking antibodies detailed above (see *Antibodies and chemicals*).

The blocking agent mevastatin was preincubated with the target cells at a concentration of 25 μmol/L for 2 hours at 37°C. The cytotoxicity assay was conducted in the presence of 25 μmol/L mevastatin. Experiments with zoledronate were performed depending on the target cells. BCSC culture cells were preincubated with 10 μmol/L zoledronate over night at 37°C and washed before the cytotoxicity assay. In contrast, for experiments including xenograft-derived tumor cells, all target cells were preincubated with 10 μmol/L zoledronate for 2 hours and the cytotoxicity assay was performed in the presence of 10 μmol/L zoledronate.

To analyze the effect of IFNα 2B, target cells were preincubated with 2 × 10^3^ U or 2 × 10^4^ U IFNα 2B (Stemcell, 78077) for 1 hour at 37°C. The assays were conducted with a final concentration of 1 × 10^3^ U or 1 × 10^4^ U IFNα 2B.

To analyze γδ T-cell degranulation, BCSCs or xenograft-derived tumor cells were labeled with 20 μmol/L cell proliferation dye eFluor450 (Thermo Fisher Scientific). A total of 1 × 10^5^ tumor cells and 1 × 10^5^ γδ T cells were cocultured for 3 hours at 37°C in the presence of 1 μL anti-CD107a-PE (BD Biosciences). Medium or stimulation with anti-CD3 and anti-CD28 (both 1 μg/mL; plate-bound) served as negative and positive controls, respectively. Cells were harvested and analyzed by flow cytometry.

### IFN**γ** ELISA

A total of 1 × 10^5^ γδ T cells were cocultured with tumor cells for 24 hours at a ratio of 1:1. Target cells were preincubated with 20 μmol/L zoledronate for 2 hours or with 2 × 10^3^ U or 2 × 10^4^ U IFNα 2B for 1 hour at 37°C before coculturing them with γδ T cells. The assay was conducted with a final concentration of 10 μmol/L zoledronate or 1 × 10^3^ U or 1 × 10^4^ U IFNα 2B. The culture supernatant was then analyzed for secreted IFNγ using an IFNγ ELISA kit (Thermo Fisher Scientific, EHIFNG) according to the manufacturer's instructions.

### Xenograft tumor model and **γδ** T-cell treatment

NOD SCID mice (NOD.CB17-Prkdcscid/Rj, Janvier Labs) and Rag2-γ-mice (Rag2tm1.1Flv IL-2rgtm1.1Flv, Jackson Laboratory) were housed at the Center for Experimental Models and Transgenic Service, Freiburg, under specific pathogen-free conditions using individually ventilated cages. Mouse handling and experiments were performed in accordance with German Animal Welfare regulations and approved by the Regierungspräsidium Freiburg (animal protocols G17/137 and G19/168).

The orthotopic transplant was performed as described previously (22). A total of 5 × 10^5^ BCSCs were mixed with 1 × 10^6^ irradiated HDF in a 1:1 mixture of MSC medium and Matrigel (growth factor reduced, Corning) and transplanted into each fat pad of the two #4 mammary glands of female NOD SCID mice. Mice were anesthetized during the procedure using an isoflurane inhalator. Before treatment started, mice were randomized into three groups: vehicle (PBS), γδ T cell, γδ T cell MMP14. Treatment was initiated for each mouse individually at a tumor volume of at least 4 mm^3^ as indicated for each experiment. A total of 5 × 10^6^ γδ T cells (culture purity ≥95%; <5% Vδ1^+^) were intravenously injected three times per week for the indicated time period. In addition, mice received 0.6 × 10^6^ IU of IL2 (Proleukin, Novartis) in incomplete Freund's adjuvant subcutaneously in the abdomen to support γδ T-cell survival *in vivo*. Checkpoint inhibition using the α-PD-1 antibody Nivolumab (Opdivo, Bristol Myers Squibb) and a corresponding α-human IgG4κ isotype control (Hölzel Diagnostika Handels GmbH, HG4K-25) was performed by additional biweekly intraperitoneal injections of 200 μg of the respective antibody. Tumor sizes were defined by caliper measurement. Tumor volumes were calculated using the formula *V* = 4/3 × π × *r*^3^.

### Preparation of tumor single-cell suspensions

Tumors from BCSC5 orthotopic xenografts (see *Xenograft tumor model)* were cut into small pieces using a razor blade and digested with 1 mg/mL collagenase IV (Sigma-Aldrich, C5138) and 0.1 mg/mL DNAse I (Roche, 37770400) at 37°C for 45 minutes. The digested tissue was filtered through 70 and 40 μm filters. To remove remaining cell debris, the cell suspension was cleared via centrifugation through a FBS layer. Erythrocytes were removed using Ammonium-Chloride-Potassium (ACK) lysis and recovered cells were used for experiments when the proportion of human EpCAM-positive (EpCAM^+^) cells was > 95%. For flow cytometric analysis, EpCAM^+^ cells were gated.

### Preparation of murine blood and liver samples

Leukocytes were isolated from blood samples by repeated erythrocyte lysis steps using ACK lysis buffer. When only minimal residual erythrocytes were left in the sample, cells were used for flow cytometric analysis. To analyze liver-derived lymphocytes, liver tissue was cut into small pieces using scissors and further dissociated through a 70 μm filter. The cell suspension was centrifuged at 60 × *g* for 1 minute at room temperature without break and the resulting supernatant was centrifuged at 850 × *g* for 8 minutes at room temperature. The pellet was then resuspended in 10 mL 37.5% Percoll (Sigma-Aldrich, P4937) in PBS and 100 U/mL heparin (Sigma-Aldrich, H3149) and centrifuged at 850 × *g* for 20 minutes at room temperature without break. Erythrocytes were removed from the pellet using ACK lysis and recovered cells were used for flow cytometry.

### Preparation of viable BCSC5 xenograft tumor slices and confocal imaging of **γδ** T-cell migration

Tumor slices were prepared as described previously (18). Briefly, BCSC5 xenograft-derived tumors were cut into small pieces using a razor blade. Tumor pieces were embedded in a 5% low-gelling-temperature agarose (Sigma-Aldrich, A9045) solution (w/v in PBS). After agarose solidification at 4°C, agarose blocks were fixed on the specimen disk of a vibratome using nontoxic tissue adhesive (3M Vetbond, 1469c). The embedded tissue was cut into 350-μm-thick slices in ice-cold PBS. Tumor slices were transferred onto 30 mm organotypic culture insert (Merck), which had been placed in the wells of a 6-well plate filled with 1.1 mL phenol red–free RPMI medium (Thermo Fisher Scientific, 11835030) supplemented with 10% FBS, 100 μg/mL penicillin, and 100 μg/mL streptomycin (slice assay medium).

For fluorescent labeling of the tumor tissue and plating of γδ T cells, prewet stainless steel flat washers were placed onto the agarose surrounding each tumor slice and the slices were incubated at 37°C for 10 minutes. Then, tumor slices were stained with α-EpCAM-BV421 (10 μg/mL) and anti-fibronectin (3.5 μg/mL) for 15 minutes at 37°C and subsequently washed with slice assay medium. A total of 1 × 10^6^ γδ T cells were labeled with 0.5 μmol/L CellTracker Green CMFDA Dye (Thermo Fisher Scientific) according to the manufacturer's instruction and mixed with an anti-rabbit-AlexaFluor564 (10 μg/mL). The solution was added on the tumor slices and incubated for 30 minutes at 37°C. Subsequently slices were washed and incubated at 37°C for 10 minutes until imaging.

Image acquisition was performed at 37°C in slice assay medium with a LSM 880 inverted laser scanning confocal microscope (Zeiss) equipped with a 25× objective (LD LCI Plan-Apochromat, NA 0.8, WD 0.57 mm, water immersion) using the Fast Airyscan mode. BV421, CMFDA, and AlexaFluor546 were excited with a 405, 488, or 561 nm laser, respectively. Nine optical planes spanning a total depth of 63 μm in the Z dimension were captured every 30 seconds for 20 to 45 minutes.

Airyscan data were first processed and stitched using the Zeiss Zen Black edition 3.0 SR. Then, ECM regions were manually defined with the help of the fibronectin staining in each individual plane along the *Z* axis. Areas negative for fibronectin signal were considered as tumor regions. Further data analysis was performed using Python and the scikit-image library. Images were first corrected for sample drift by detecting matching features in subsequent frames of the ECM channel and estimating transformation parameters based on their coordinates. Cells were segmented by intensity after background correction to reduce bleed-through of signal from the ECM channel and median filtering for smoothing. Detected features were filtered by size to remove noise and cell clumps. To facilitate tracking, cells that were apparent in more than one Z plane were only considered in the plane in which their size was maximal. Subsequent tracking of the detected cells was performed using Trackpy, and two tracks were merged if their respective initial or final point were less than 10 μm and less than two frames apart. Only tracks with data for at least six frames were considered for further analysis.

For localization analysis, cells were considered to localize to tumor regions when no pixel of the γδ T-cell CMFDA signal overlapped with the fibronectin signal. A cell was defined to have a tumor dwell time >50% if it spent at least 50% of its observed frames localized to tumor regions. For speed analysis, the momentary speed of a cell was defined as the distance travelled between two successive observations of the same cell, divided by the time between these observations.

Representative microscopy images and videos were generated using Imaris 9.3.1.

### Preparation of tumor cell-derived conditioned medium

For transwell migration assays, conditioned medium (CM) was generated by harvesting the supernatant of BCSC cultures after 5 days. Culture supernatants were cleared from residual cells by centrifugation and stored at −80°C until use.

To generate CM from xenograft-derived cells, 5 × 10^6^ tumor cells were cultured in γδ T-cell medium for 24 hours at 37°C in a humidified atmosphere of 5% CO_2_. Culture supernatants were cleared from residual cells by centrifugation and directly used for experiments.

### Transwell and matrigel migration assays

For transwell migration assays toward CM, the CM was diluted 1:1.67 in γδ T-cell medium/1% FBS. A total of 250 μL were transferred to the receiver wells of a 96-well transwell plate (Corning). Medium without cells, incubated in the same conditions as BCSCs, served as negative control. For migration toward the chemokine CXCL12, γδ T-cell medium/1% FBS was supplemented with 50 ng/mL CXCL12 (PeproTech, 300-28A).

For transwell assays toward tumor cells, tumor cells were labeled with 20 μmol/L cell proliferation dye eFluor450 (Thermo Fisher Scientific). A total of 1.5 × 10^5^ tumor cells in 250 μL γδ T-cell medium/1%FBS were seeded into the receiver wells of a 96-well transwell plate. γδ T-cell medium/1% FBS served as negative control.

A total of 2 × 10^5^ γδ T cells in 100 μL γδ T-cell medium/1% FBS were seeded in the well of the permeable support with 5-μm pore size (Corning). After 3 hours incubation at 37°C, transmigrated cells were stained to distinguish T-cell subsets and counted via flow cytometry.

Matrigel migration assays were performed as described previously (25, 26). Briefly, 3.5 × 10^4^ γδ T cells were resuspended in 10 μL ice-cold Matrigel (Corning, 354234) and plated into the inner well of a precooled μ-Slide Angiogenesis (ibidi). After cell settling at 4°C, samples were solidified at 37°C. A total of 15 μL Matrigel were added on top of the cell-containing gels. Following solidification at 37°C, wells were filled with γδ T-cell medium supplemented with 10% FBS, 30 U/ml IL2, and 50 ng/mL CXCL12. After 48 hours, samples were fixed with 4% paraformaldehyde/0.25% glutaraldehyde for 20 minutes at room temperature. Gels were washed with PBS and cells were permeabilized with 0.5% Triton-X100 (Carl Roth) in PBS for 40 minutes. After washing, nuclei were stained with 5 μg/mL Hoechst 33342 (Sigma-Aldrich, 14533) for 3 hours in the dark and samples were imaged using confocal microscopy.

Images were acquired using a C2 confocal microscope (Nikon) with a 20× objective (NA: 0.75, WD: 1 mm). Hoechst 33342 was excited with a 405 nm laser. Z stacks with a step size of 2 μm were imaged. Quantification of the migration distance was performed by using Python and the scikit-image library. Briefly, the voxels of the stacks were segmented into foreground (cells) and background by intensity thresholding. Overlapping cells were then separated in 3D with the Watershed algorithm. The position of a cell was defined to coincide with its centroid. Migration distances were normalized between images by baseline subtraction per image: the baseline migration distance for an image was defined as the 10th percentile of all cells’ Z coordinates. This value was subtracted from all Z coordinates. Z coordinates were set to 0 if they became negative after baseline correction.

### Proteomics

#### Sample preparation

BCSC5 culture cells and xenograft-derived tumor cells were washed with PBS, pelleted, frozen in liquid nitrogen, and stored at −80°C until further processing. Cell pellets were lysed in 400 μL lysis buffer [4% sodium dodecyl sulfate, 50 mmol/L tetraethylammonium bromide (pH 8.5), and 10 mmol/L tris(2-carboxyethyl)phosphine hydrochloride]. Lysates were boiled for 5 minutes and then sonicated for 15 minutes at high intensity (30 seconds on/30 seconds off). After sonication, DNA and RNA were degraded using Benzonase endonuclease (Sigma/Merck, E1014). The protein concentration was measured with EZQ Protein Quantitation Kit (Thermo Fisher Scientific, EZQ R33200). Lysates were alkylated in the dark with 20 mmol/L iodoacetamide for 1 hour at room temperature. For protein clean-up, 200 μg SP3 paramagnetic beads were added to the lysates, and proteins were bound to the beads by adding acetonitrile with 0.1% formic acid. Beads were washed in 70% ethanol and 100% acetonitrile before elution in digest buffer [0.1% sodium dodecyl sulfate, 50 mmol/L tetraethylammonium bromide (pH 8.5), and 1 mmol/L CaCl_2_] and digested sequentially with LysC (Wako), then Trypsin (Promega, V5280), each at a 1:100 w/w (enzyme:protein) ratio. Peptide clean-up was performed according to the SP3 protocol.

#### Tandem mass tag labeling and basic C18 reverse phase chromatography fractionation

Each sample (200 μg of peptides each) was resuspended in 100 μL of 100 mmol/L tetraethylammonium bromide buffer. TMT 10-plex (Thermo Fisher Scientific, 90110) labeling was performed according to manufacturer's protocol. To ensure complete labelling, 1 μg of labeled samples from each channel was analyzed by LC/MS-MS prior to pooling. The mixture of TMT 10-plex sample was desalted with Sep Pak C18 cartridge (Waters), and then fractionated by basic C18 reverse phase chromatography as described previously (27).

#### LC/MS-MS analysis

The LC separations were performed as described (27) with a Thermo Dionex Ultimate 3000 RSLC Nano liquid chromatography instrument. Approximately 1 μg of concentrated peptides (quantified by NanoDrop) from each fraction were separated over an EASY Spray column (C18, 2 μm, 75 μm × 50 cm) with an integrated nanoelectrospray emitter at a flow rate of 300 nL/minute. Peptides were separated with a 180-minute segmented gradient. Eluted peptides were analyzed on an Orbitrap Fusion Lumos (Thermo Fisher Scientific) mass spectrometer.

#### Data analysis

All the acquired LC/MS-MS data were analyzed using Proteome Discoverer software v.2.2 (Thermo Fisher Scientific) with Mascot search engine. A maximum missed cleavages for trypsin digestion was set to 2. Precursor mass tolerance was set to 20 ppm. Fragment ion tolerance was set to 0.6 Da. Carbamidomethylation on cysteine and TMT 10-plex tags on N termini as well as lysine (+229.163 Da) were set as static modifications. Variable modifications were set as oxidation on methionine (+15.995 Da) and phosphorylation on serine, threonine, and tyrosine (+79.966 Da). Data were searched against a complete UniProt Human (Reviewed 20,143 entries downloaded Nov 2018). Peptide spectral match error rates with a 1% FDR were determined using the target-decoy strategy coupled to Percolator modeling of true and false matches.

Both unique and razor peptides were used for quantitation. Signal-to-noise (S/N) values were used to represent the reporter ion abundance with a coisolation threshold of 50% and an average reporter S/N threshold of 10 and above required for quantitation from each MS3 spectra to be used. The summed abundance of quantified peptides were used for protein quantitation. The total peptide amount was used for the normalisation. Protein ratios were calculated from medians of summed sample abundances of replicate groups. SD was calculated from all biological replicate values. The standard deviation of all biological replicates lower than 25% was used for further analyses.

Differentially regulated proteins were identified using a linear-based model (limma) on the normalized log_2_ protein abundance. *P* value < 0.05 threshold was used as significance threshold. The Generally Applicable Gene-set Enrichment was used to retrieve the enriched processes. Several databases from MSigDB were used including Hallmark, Reactome, Gene Ontology, and immunologic signatures gene sets. *P* < 0.05 was used as significance threshold.

### IP and Western blot analysis

Cells or xenografted tumors were lysed for 20 minutes on ice in lysis buffer [0.1% Nonidet P-40, 50 mmol/L HEPES (pH 7.0), 250 mmol/L NaCl, 5 mmol/L EDTA, 1 mmol/L phenylmethylsulfonyl fluoride, and 0.5 mmol/L dithiothreitol]. Lysis was followed by a 15-minute centrifugation to pellet the nuclei and insoluble materials. The supernatants were subsequently used as indicated. For HMG-CoA reductase (HMGCR) IP, 2 μg of anti-HMGCR antibody together with 10 μL of a mixture of 1:1 protein A and G coupled sepharose beads (GE Healthcare, 17513801 and 17061801) were added to lysates and incubated overnight at 4°C. Proteins from lysate or IP were subjected to SDS-PAGE followed by immunoblotting according to standard procedures (see *Antibodies and chemicals*). Protein bands were detected by chemiluminescence under a CCD camera (ImageQuant LAS 4000; GE Healthcare). Relative band intensity was quantified by ImageJ software and ImageQuantTL software (GE Healthcare).

### HMGCR activity assay

The enzymatic activity of HMGCR was evaluated by quantitation of the NADPH extinction using the HMG-CoA reductase assay kit (Sigma-Aldrich, CS1090) with the samples generated for Western blot analysis using 50 μg total protein per reaction. A recombinant HMGCR provided by the kit was used as a positive control, the recombinant enzyme incubated with 5 μL of the Pravastatin (provided in the kit) was used as negative control, and only lysis buffer was used as blank. Samples, buffers, and substrates were added following the order in the manufacturer's protocol. Absorbance was measured at 340 nm using a microplate reader. The activity is expressed as AU/mg protein where 1 unit (AU) is the amount of HMGCR oxidating 1 μmol of NADPH in a minute at 37°C.

### Statistical analysis

All data were tested for normality applying the D'Agostino and Pearson or Shapiro–Wilk test. For the comparison of two groups, an unpaired two-tailed Student *t* test was applied. For data not meeting the criteria for normality, the Mann–Whitney or Wilcoxon signed-rank test was applied. For the analysis of more than two groups, one-way ANOVA was applied in case of normally distributed data. Correction for multiple comparisons was performed by Dunnett test (comparing all groups with one control group). Nonparametric data were analyzed using the Kruskal–Wallis test followed by Dunn test (comparing all groups with one control group) to correct for multiple comparisons. Grouped analyses were tested using two-way ANOVA. To correct for multiple comparisons, Dunnett (comparing groups with respective control group inside of one row), Sidak (comparing groups with respective control group inside of one column or comparing two groups in one row), or Tukey (comparing all groups with each other) test was applied. If data were analyzed compared with a hypothetical value of 1 or 100, we used the one-sample *t* test for normally distributed data and the one-sample Wilcoxon test when nonparametric testing was suggested. log-rank test (Mantel–Cox) was used to calculate significance of differences between survival curves. Statistical analysis was performed using GraphPad Prism (v9, GraphPad Software). Applied analyses and statistical significances are indicated in the corresponding figures and figure legends. All data are represented as means ± SEM. Differences with *P* ≤ 0.05 were considered statistically significant. ns, nonsignificant; *, *P* ≤ 0.05; **, *P* ≤ 0.01; ***, *P* ≤ 0.001; ****, *P* ≤ 0.0001. All data values and the corresponding statistical tests of each graph are available.

### The Cancer Genome Atlas Analysis

The Cancer Genome Atlas (TCGA) RNA sequencing (RNA-seq) data were downloaded from TCGAbiolinks R/Bioconductor package. Only primary tumor samples from TNBC were retained in the analysis. In total, 180 RNA-seq samples with survival data available were analyzed. A list of TCGA barcodes is provided in Supplementary Table S1. Downstream analysis was performed with R (v4.2.2). Read counts were normalized by the library size (count per million). Genes quantified in less than 75% of the dataset were filtered out. Survival analysis, including Cox proportional hazard, was performed with “survival” and “survminer” R package.

### Data availability

The proteomic data generated in this study are publicly available via ProteomeXchange with identifier PXD039463. Other data generated in this study are available within the article and its Supplementary Data or from the corresponding author upon reasonable request.

## Results

### Expanded **γδ** T cells efficiently kill patient-derived triple-negative BCSCs

To test the cytotoxic potential of human γδ T cells toward patient-derived triple-negative BCSCs, we expanded γδ T cells from PBMCs of healthy donors using concanavalin A (ConA) stimulation. As described previously (23), expansion resulted in a specific enrichment of effector memory Vδ1^+^ and Vδ2^+^ T cells (Supplementary Fig. S1A). The expression of the activating natural killer–cell receptor NKG2D, which mediates the cytolytic activity of γδ T cells (28), was upregulated during γδ T-cell expansion (Supplementary Fig. S1B). We observed significantly higher NKG2D expression in Vδ2^+^ compared with Vδ1^+^ T cells during expansion; however, NKG2D expression was comparable at the end of the culture period (28 days). It has been reported that the overt expansion of αβ T cells, their genetic modification or their stimulation after transfer to a patient can result in T-cell exhaustion and functional failure (29). Therefore, we followed the expression of the inhibitory receptors PD-1, TIM-3, and LAG-3 throughout the expansion (Supplementary Fig. S1B). PD-1 was upregulated within the first 10 days after ConA stimulation and then declined to basal levels. TIM-3, in contrast, exhibited a constant increase in expression as the expansion progressed. LAG-3 expression was drastically increased in the first 10 days after ConA stimulation, was then reduced but remained upregulated until the end of the expansion. The three inhibitory receptors were expressed at significantly higher levels in Vδ2^+^ compared with Vδ1^+^ T cells upon stimulation, and TIM-3 and LAG-3 remained highly expressed in Vδ2^+^ T cells at the end of the observed expansion period. Of note, the percentage of Vδ1^+^ and Vδ2^+^ T cells strongly varied between healthy donors. In the majority of donors, Vδ2^+^ T cells were highly enriched (Supplementary Fig. S1C). Expanded γδ T cells were used for experiments between day 14 and day 35 after starting the expansion because the cells have reached a stable phenotype at this time window and cytotoxicity remained stable (Supplementary Fig. S1D).

**Figure 1.**
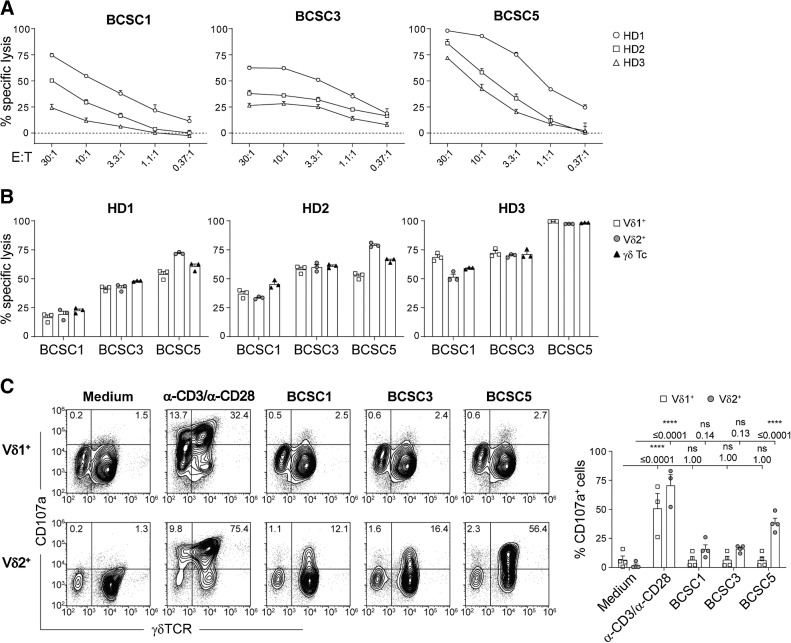
Expanded γδ T cells recognize and kill BCSCs *in vitro*. **A,***In vitro* killing of luciferase-expressing BCSCs by γδ T cells after 8 hours at various effector to target (E:T) ratios. Results from two independent experiments with a total of 3 healthy donors (HD) of γδ T cells are shown (means ± SEM). **B,***In vitro* killing of BCSCs by γδ T cells and MACS-separated Vδ1^+^ and Vδ2^+^ T cells performed as in **A**. Cells were cocultured at an E:T ratio of 10:1. Results from 3 healthy donors of γδ T cells are shown (means ± SEM). **C,** Flow cytometry-based analysis of degranulation by MACS-separated Vδ1^+^ or Vδ2^+^T cells in response to BCSC contact for 3 hours. Stimulation with anti-CD3 and anti-CD28 served as positive control. Representative dot plots (left) and statistical analysis (right) of the percentage of CD107a^+^Vδ1^+^ or CD107a^+^Vδ2^+^cells. Results from 2 healthy donors of γδ T cells obtained in three independent experiments were pooled (means ± SEM). Two-way ANOVA followed by Sidak *post hoc* test comparing stimulated or cocultured cells with the corresponding medium control. ****, *P* ≤ 0.0001. E:T, effector to target; HD, healthy donor; ns, nonsignificant.

Next, we determined whether these γδ T cells could kill patient-derived triple-negative BCSCs *in vitro*. Expanded γδ T cells from 3 healthy donors killed all tested patient-derived triple-negative BCSC lines (BCSC1, BCSC3, and BCSC5) in an effector to target ratio-dependent manner (Fig. 1A). BCSC5 was most efficiently killed. To test whether the cytotoxicity was mediated by Vδ1^+^ or Vδ2^+^ T cells, we separated these two T-cell subsets after expansion (Supplementary Fig. S1D). Both Vδ1^+^ and Vδ2^+^ T cells displayed similar cytotoxicity in response to BCSC1, BCSC3, and BCSC5 (Fig. 1B). BCSCs only induced degranulation in Vδ2^+^ T cells, measured indirectly via CD107a (Fig. 1C). This was most prominent for BCSC5, which led to significantly higher degranulation in Vδ2^+^ T cells compared with BCSC1 and BCSC3. That Vδ1^+^ T cells did not degranulate after BCSC contact was not a consequence of a general incapability of Vδ1^+^ T cells to degranulate because stimulation with α-CD3 and α-CD28 antibodies led to CD107a accumulation in both T-cell subsets (Fig. 1C). These results suggest that Vδ1^+^ and Vδ2^+^ T cells kill patient-derived human BCSCs using different mechanisms.

### Expanded **γδ** T cells are attracted by BCSC CM

**Figure 2.**
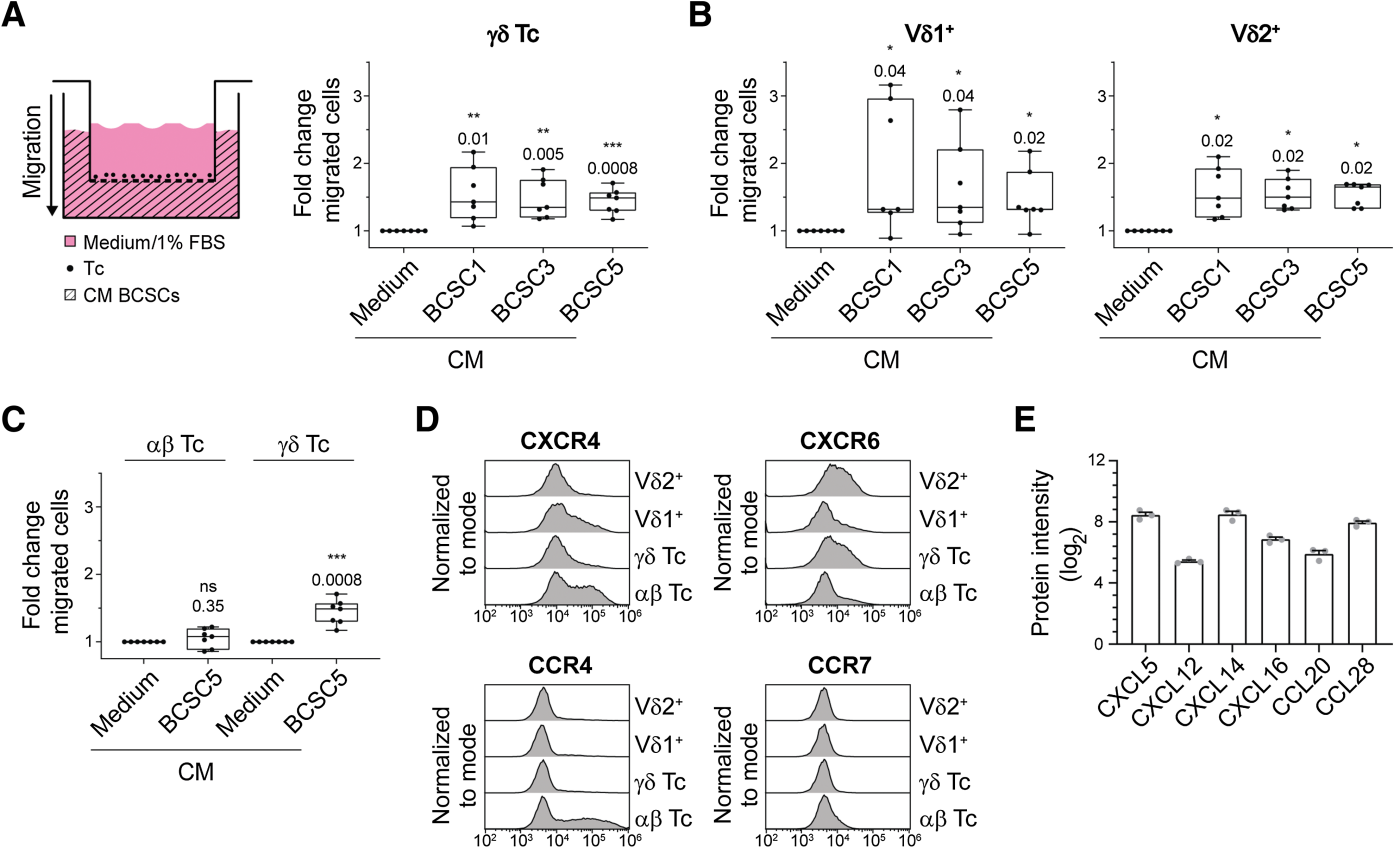
Expanded γδ T cells migrate toward BCSC CM. **A,** Schematic transwell migration assay (left). Primary T cells were seeded in the wells of a permeable support with 5-μm pore size. The lower compartment was filled with BCSC CM and cells that transmigrated into the lower compartment were analyzed by flow cytometry. Statistical analysis of transmigrated γδ T cells (CD3^+^γδTCR^+^) is shown (right). Basal migration toward medium was set to 1.0 and fold changes from two independent experiments using 7 healthy donors in total were pooled (median, minimum to maximum). One-sample *t* test against hypothetical value of 1.0. **B,** Transwell assays were performed and analyzed as in **A**. Transmigrated Vδ1^+^ (CD3^+^γδTCR^+^Vδ1^+^) and Vδ2^+^ (CD3^+^γδTCR^+^Vδ2^+^) T cells were distinguished by flow cytometry. One-sample *t* test (for Vδ1^+^) or one-sample Wilcoxon test (for Vδ2^+^). **C,** Transwell assays were performed and analyzed as in **A**. Comparison of transmigrated αβ T cells (CD3^+^γδTCR^−^) and γδ T cells (CD3^+^γδTCR^+^; data from **A**) in response to BCSC5 CM. One sample *t* test against hypothetical value of 1.0. **D,** Representative chemokine receptor expression levels in αβ (CD3^+^γδTCR^−^), γδ (CD3^+^γδTCR^+^), Vδ1^+^ (CD3^+^γδTCR^+^Vδ1^+^), and Vδ2^+^ (CD3^+^γδTCR^+^Vδ2^+^) T cells. Shown are representative flow cytometry histograms from one healthy donor out of 3 healthy donors analyzed. **E,** Chemokine expression levels determined by quantitative mass spectrometry of BCSC5. Protein intensities (log_2_) of three replicates are shown (means ± SEM). *, *P* ≤ 0.05; **, *P* ≤ 0.01; ***, *P* ≤ 0.001.

*In vivo*, in addition to recognizing and killing BCSCs, γδ T cells need to be attracted and migrate toward the tumor sites. Therefore, we next assessed the migratory capacity of γδ T cells toward BCSC CM in transwell experiments. CM from BCSC1, BCSC3, and BCSC5 efficiently attracted γδ T cells (Fig. 2A). The migration of Vδ1^+^ T cells toward BCSC CM was increased compared with control medium, but strongly dependent on the T-cell donor. In contrast, Vδ2^+^ T cells from all tested healthy donors migrated robustly in response to BCSC CM (Fig. 2B). γδ T cells outperformed αβ T cells regarding their migration ability toward BCSC5 CM (Fig. 2C).

The different migratory responses exhibited by the specific T-cell subsets were more thoroughly investigated by analyzing the expression levels of 12 chemokine receptors (CXCR1, CXCR3, CXCR4, CXCR5, CXCR6, CCR2, CCR3, CCR4, CCR5, CCR6, CCR7, and CCR10). Among those, we found four of them, namely CXCR4, CXCR6, CCR4, and CCR7, to be differentially expressed in αβ, γδ, Vδ1^+^, and Vδ2^+^ T cells (Fig. 2D). The expression of CXCR6 correlated well with the migratory responses observed. The only known ligand for CXCR6 is CXCL16, and CXCL16 was indeed detected in a proteomic approach using BCSC5 culture cells (Fig. 2E). Besides CXCL16, we identified the chemokines CXCL12, CXCL5, CXCL14, CCL20, and CCL28 to be expressed by BCSC5 (Fig. 2E). However, the chemokine expression pattern alone might be insufficient to identify the chemokine-chemokine receptor pairs involved in the attraction of T cells by BCSC CM due to the high degree of promiscuity defining the chemokine system. For example, even migration toward the well-known T-cell attractant CXCL12 only partially correlated with the expression levels of its best-characterized receptor CXCR4 (Supplementary Fig. S2A and S2B).

Taken together, ConA-expanded γδ T cells most efficiently recognized and killed BCSC5 among the tested BCSCs, and exhibited a robust migration toward BCSC5 CM. Therefore, we focused our studies on BCSC5 and conducted further experiments with γδ T-cell cultures containing mainly Vδ2^+^ T cells and less than 10% of Vδ1^+^ T cells to minimize heterogeneity.

### MMP14 expression in **γδ** T cells increases their migration capacity in ECM-rich environments

**Figure 3.**
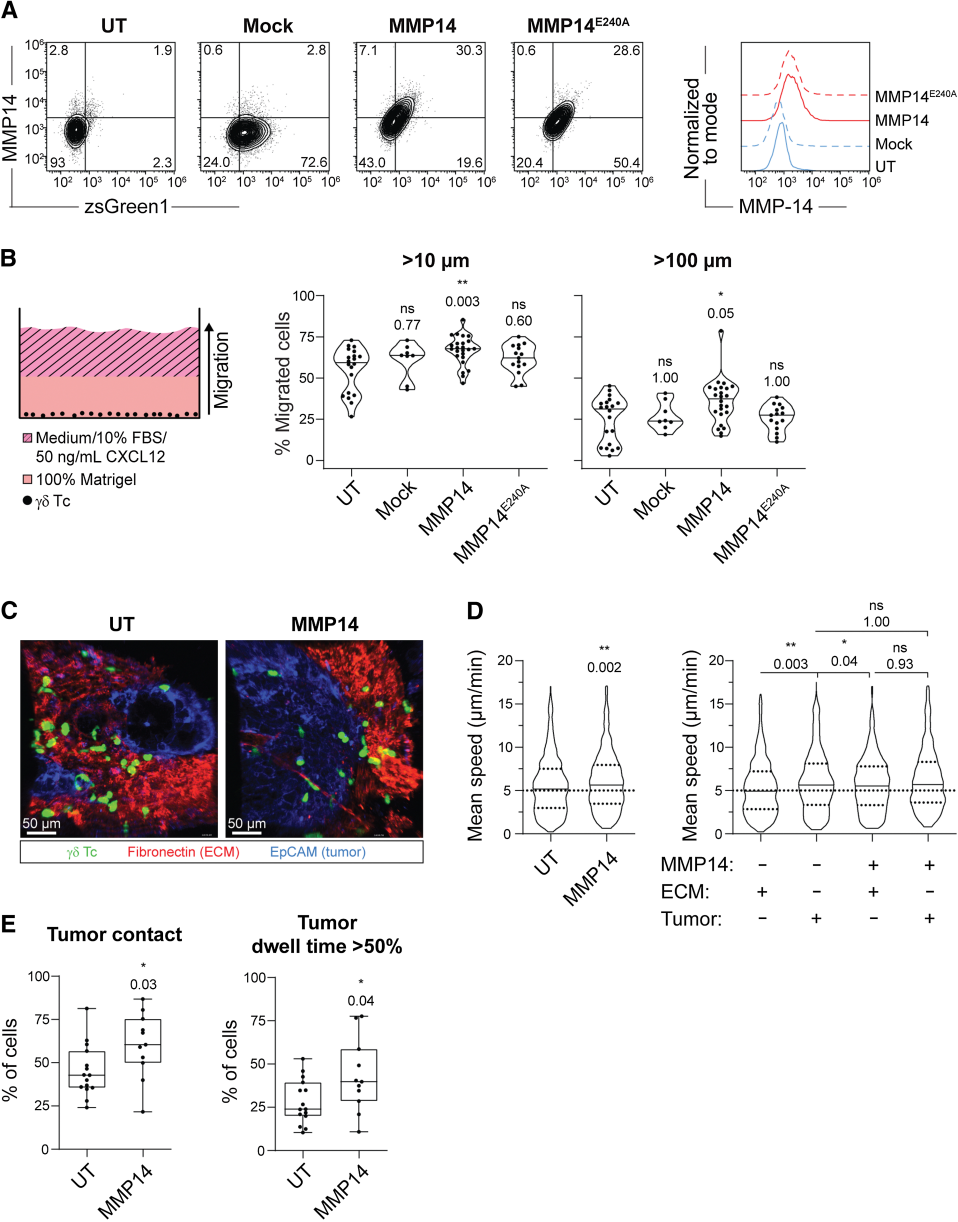
MMP14 expression increases γδ T-cell migration in basement membrane-like Matrigel and in BCSC5 tumor tissue. **A,** Representative dot plots (left) and histograms (right) of γδ T cells expressing mock, MMP14, or MMP14^E240A^ on day 3 after transduction with a MOI of 5. UT cells served as control. **B,** Schematic illustration of the 3D migration assay in Matrigel (left). γδ T cells were seeded into ibidi μ-angiogenesis slides in 100% Matrigel. Migration toward medium supplemented with 10% FBS and 50 ng/mL CXCL12 was assessed via confocal microscopy after 48 hours. Statistical analysis (right) of the percent of γδ T cells migrating further than 10 and 100 μm (medians are indicated). Results of 8–24 wells per condition from five independent experiments are shown. Kruskal–Wallis test followed by Dunn *post hoc* test comparing transduced with untransduced cells. **C,** Migration of CMFDA-labeled UT (see also Supplementary Video S1) or MMP14 expressing (see also Supplementary Video S2) γδ T cells (green) in vibratome sections of viable BCSC5 xenograft tumors. Shown are representative Z-projections of confocal time-lapse videos from BCSC5 xenograft tumor slices stained for EpCAM (blue) and fibronectin (red) to identify tumor cell regions and stromal compartments, respectively. **D,** The mean speed of γδ T cells migrating in BCSC5 xenograft tumor slices is shown (left; Mann-Whitney test). The same data were analyzed with respect to the mean speed of γδ T cells in stromal ECM compartments (fibronectin^+^) and tumor cell regions (EpCAM^+^) of BCSC5 xenograft tumor slices (right; Kruskal-Wallis test followed by Dunn *post hoc* test comparing all groups against each other). **E,** The percentage of cells, which have been able to enter EpCAM^+^ tumor tissue (left) or which have resided over 50% of their monitored time inside EpCAM^+^ tumor tissue (right), was quantified in each time-lapse experiment (median, minimum to maximum). Unpaired *t* test, two tailed. **D,** Results from 15 (UT) and 11 (MMP14) time-lapse acquisitions from seven independent experiments are shown including at least 440 tracks per group. Outliers were identified and removed using the ROUT method (*Q* = 2%). *, *P* ≤ 0.05; **, *P* ≤ 0.01; ***, *P* ≤ 0.001. ECM, extracellular matrix; EpCAM, epithelial cellular adhesion molecule; UT, untransduced.

Our transwell assays demonstrated that γδ T cells were efficiently attracted toward BCSC5 CM. However, BCSC-derived xenotransplanted tumors are surrounded by a dense ECM, similar to the original TNBC tumors (22). This ECM-rich stromal compartment might hamper γδ T-cell migration and infiltration. Therefore, we aimed to boost γδ T-cell migration by expressing the membrane-anchored MMP14 in γδ T cells. MMP14 is one of 26 known endopeptidases of the human MMP protein family (30). It can cleave a plethora of ECM proteins like fibronectin, collagen (type I, II, and III), and laminin (31, 32). Furthermore, MMP14 has been intensively studied in the process of tumor-cell migration where it was shown to possess promigratory functions (33). We found that MMP14 was endogenously expressed in γδ T cells directly after ConA stimulation but was downregulated over time. In contrast, αβ T cells did not upregulate MMP14 upon activation (Supplementary Fig. S3A). To maintain MMP14 expression, expanded γδ T cells were lentivirally transduced with a mock vector, MMP14, or the catalytically inactive mutant MMP14^E240A^, and expression was verified by flow cytometry (Fig. 3A; Supplementary Fig. S3B). We then assessed the effect of MMP14 on γδ T-cell migration towards CXCL12 and FBS in Matrigel, a model matrix for basement membranes (Fig. 3B). MMP14 expression increased the percentage of γδ T cells migrating further than 10 or 100 μm, while the catalytically inactive mutant failed to promote migration (Fig. 3B). These findings demonstrate that MMP14 has the potential to promote the migration of γδ T cells in basement membrane–like ECM.

To test whether the promigratory function of MMP14 observed in Matrigel also supports interstitial migration in tumors, we analyzed γδ T-cell migration in viable slices of BCSC5 xenograft tumors via confocal microscopy following published protocols (Supplementary Fig. S3C; ref. 18). For this purpose, BCSC5 cells were orthotopically transplanted into the mammary fat pad of NOD SCID mice as described previously (22, 34, 35). CMFDA-labeled nontransduced γδ T cells or γδ T cells expressing MMP14 were plated on top of unfixed xenograft-derived tumor slices and were microscopically monitored. EpCAM and fibronectin staining served to distinguish tumor islets and ECM-rich stroma, respectively [Fig. 3C; Supplementary Videos S1 and S2, for untransduced (UT) and MMP14, respectively]. MMP14 expression increased the interstitial migration speed of γδ T cells when compared with nontransduced cells (Fig. 3D, left). When differentially analyzing the migration speed of γδ T cells within the tumor tissue or the stromal ECM, we observed that nontransduced γδ T cells migrated faster in the tumor tissue than in the stromal ECM (Fig. 3D). This is in line with observations made for αβ T cells in ovarian and lung cancer (19). MMP14 expression increased the average speed of γδ T cells in the ECM to the speed exhibited in the tumor tissue, supporting the functional role of MMP14 in cleaving the tumor-associated ECM (Fig. 3D, right). In addition, a higher percentage of cells per time-lapse experiment was in direct tumor contact when the cells expressed MMP14 (Fig. 3E) and a bigger fraction of γδ T cells resided in the tumor for more than 50% of their monitored time when expressing MMP14 (Fig. 3E, right). Altogether, these results suggest that the protease MMP14 can boost the migration of γδ T cells in the ECM-enriched tumor environment, and might thereby help to overcome limitations that hamper γδ T-cell migration *in vivo* and, ultimately, therapy outcome.

### **γδ** T cells fail to control BCSC5 orthotopic xenografts

**Figure 4.**
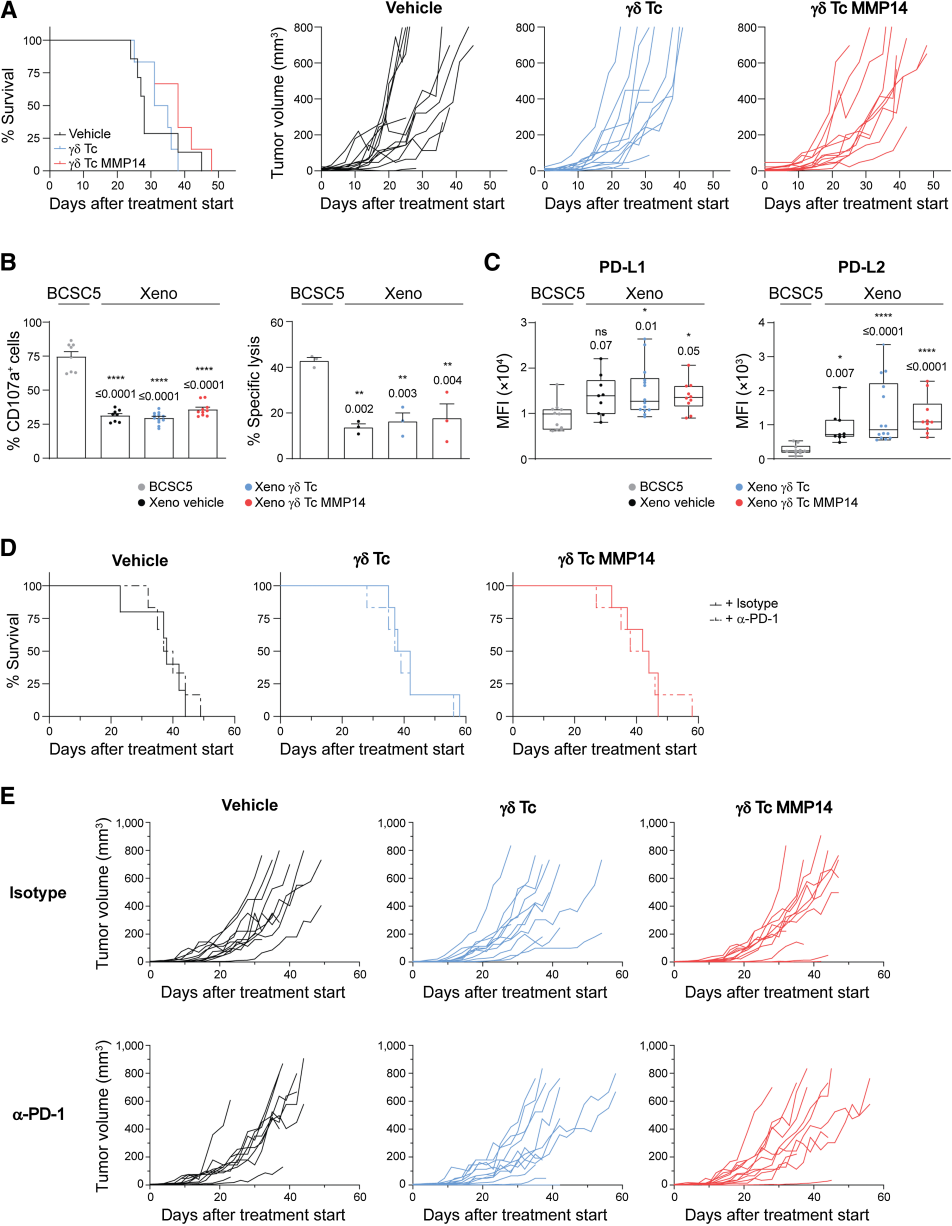
γδ T cells fail to control BCSC5 xenografts in NOD SCID mice. **A,** Kaplan–Meier plot (left) of BCSC5 xenograft-bearing mice upon treatment with γδ T cells (blue), γδ T cells expressing MMP14 (red) or vehicle control (black; *n* = 6–7 mice per group). Differences were not statistically significant, log-rank test (Mantel-Cox). BCSC5 tumor growth curves (right) for individual mice after treatment start. **B,** γδ T cell-mediated degranulation (left) and cytotoxicity (right) of BCSC5 culture cells or xenograft-derived tumor cells (xeno). Xenograft-bearing mice were treated with γδ T cells, γδ T cells expressing MMP14 or vehicle. For the degranulation assay, γδ T cells were cocultured with the respective tumor cells for 3 hours. The percentage of CD107a^+^ cells of Vδ2^+^-gated cells is shown (means ± SEM). Four to six tumors were analyzed per group and each tumor was tested with 2 healthy donors of γδ T cells. For *in vitro* killing of ^51^Cr-labeled tumor cells, γδ T cells were cocultured with the target cells for 5 hours at an E:T ratio of 30:1. Results from three independent experiments with a total of 3 healthy donors of γδ T cells and three tumors per group were pooled (means ± SEM). One-way ANOVA followed by Dunnett *post hoc* test comparing xenograft with culture cells. **C,** Flow cytometry-based analysis of PD-L1 and PD-L2 expression levels in xenograft-derived EpCAM^+^ tumor cells (median, minimum to maximum). Mean fluorescence intensity (MFI) of 9–14 tumors per group is shown. Kruskal–Wallis test followed by Dunn *post hoc* test comparing xenograft with culture cells. **D,** Kaplan–Meier plots of NOD SCID mice upon treatment with vehicle control (black), γδ T cells (blue) or γδ T cells expressing MMP14 (red) in combination with an anti–PD-1 (nivolumab) or isotype control antibody (*n* = 5–6 per group). Treatment start was defined for each mouse individually when the first tumor reached a volume of at least 4 mm^3^. A total of 5 × 10^6^ γδ T cells were injected intravenously three times per week. In addition, mice received 0.6 × 10^6^ IU IL2 (Proleukin S) on the day of treatment start and every 21 days until the end of the experiment. The end of the experiment was defined by a tumor volume of 800 mm^3^. No significant differences were obtained, log-rank (Mantel-Cox) test. **E,** BCSC5 tumor growth curves for individual mice upon treatment with γδ T cells (blue), γδ T cells expressing MMP14 (red) or vehicle control (black) in combination with anti–PD-1 or isotype control antibody (*n* = 5–6 per group). *, *P* ≤ 0.05; **, *P* ≤ 0.01; ***, *P* ≤ 0.001.

After establishing a system to maximize γδ T-cell targeting of BCSC *ex vivo*, we assessed whether γδ T cells could control BCSC5 tumor growth *in vivo*. To this end, we generated BCSC5 orthotopic mammary gland xenografts in NOD SCID mice as described previously (22, 34, 35). Mice were treated with a vehicle control, γδ T cells, or γδ T cells expressing MMP14 once the tumors had reached a volume of at least 4 mm^3^ (Supplementary Fig. S4A). The median survival of the mice after treatment started increased from 28 days for vehicle-treated animals to 33 and 38 days for γδ T cell-treated or γδ T cell MMP14-treated mice, respectively (Fig. 4A). Although a clear tendency toward increased survival times of the mice treated with γδ T cells or γδ T cells expressing MMP14 was observed, there was no significant increase in the overall survival between the different treatment groups. Furthermore, the treatment did not significantly affect the growth kinetics of the individual tumors, although a higher proportion of tumors slowed down their growth when MMP14 was expressed in γδ T cells (Fig. 4A, right). In addition, neither treatment with γδ T cells nor with γδ T cells expressing MMP14 increased tumor cell invasion or metastasis when compared with vehicle-treated animals. Similar results were obtained using alternative immunocompromised mice, namely Rag2^−/−^γc^−/−^ (Supplementary Fig. S4B).

These results raised the question as to whether BCSC5 xenograft-derived tumor cells could still be recognized and killed by γδ T cells. Thus, we isolated tumor cells from vehicle-treated, γδ T cell-treated or γδ T cell MMP14-treated mice after tumor resection and analyzed the degranulation capacity of γδ T cells in response to these xenograft-derived tumor cells (indicated as “xeno” in the figures). We observed that the proportion of degranulating γδ T cells was significantly reduced from 75% to approximately 30% when compared with the response toward *in vitro* cultured BCSC5 (Fig. 4B, left). Similarly, we observed reduced γδ T cell–mediated killing of xenograft-derived tumor cells compared with BCSCs from the culture (Fig. 4B, right). These effects were inherent to the tumor cells and not influenced by the γδ T-cell treatment of the tumor-bearing mice (Fig. 4B). We also determined whether secreted soluble molecules decreased the response of γδ T cells to xenograft-derived tumor cells. We analyzed γδ T-cell viability and γδ T-cell functionality after culturing the cells in CM from BCSC5 culture cells or xenograft-derived tumor cells for 24 hours as described previously (17). γδ T-cell viability was not influenced by CM from either cell type (Supplementary Fig. S4C). Likewise, neither degranulation nor the cytotoxic response of γδ T cells to BCSC5 was affected by the CM (Supplementary Fig. S4D and S4E). These findings indicate that the reduced γδ T-cell function in response to xenograft-derived tumor cells was not mediated by the secretion of soluble immunosuppressive molecules.

As mentioned before, triggering of inhibitory receptors such as PD-1 by their ligands can result in the functional inhibition of T cells. We observed that the ligands for the inhibitory receptor PD-1, PD-L1, and PD-L2, were upregulated on xenograft-derived tumor cells (Fig. 4C). To assess whether PD-1 blockade improved γδ T-cell treatment *in vivo*, we combined the adoptive transfer of γδ T cells with biweekly applications of a clinically relevant anti–PD-1 antibody (nivolumab). However, the combination treatment did not improve the overall survival of the xenograft-bearing mice but showed a slight reduction in the tumor growth kinetics *in vivo* (Fig. 4D and E). These results suggest that blocking PD-1 was not sufficient to significantly induce γδ T cell–mediated tumor rejection and that other mechanisms might be involved in the immune escape of BCSC5-derived xenografts.

### γδ T cells are efficiently attracted by xenograft-derived tumor cells

**Figure 5.**
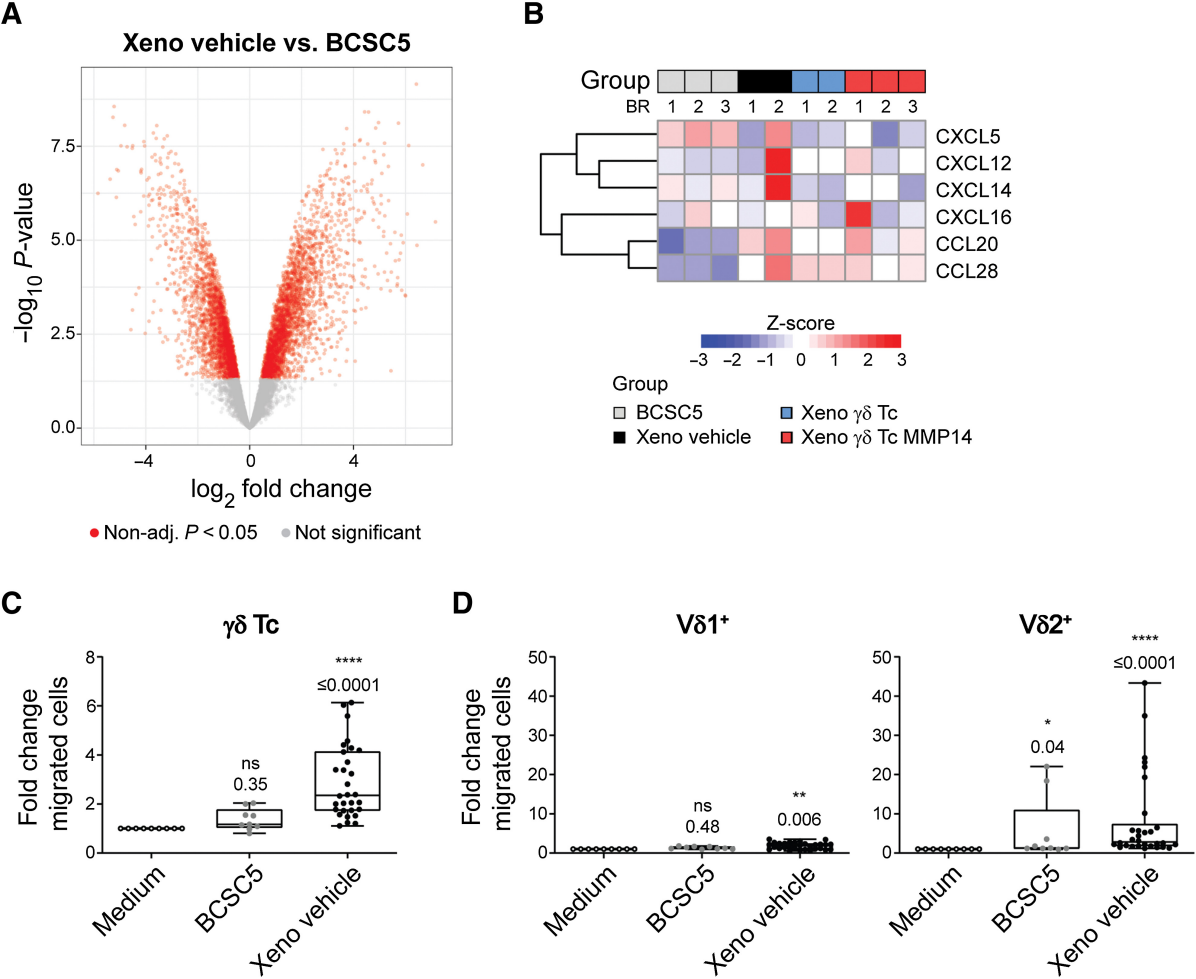
BCSCs phenotypically change *in vivo*, but still induce γδ T-cell migration. **A,** The proteomes of BCSC5 culture cells or freshly isolated xenograft-derived tumor cells were analyzed by mass spectrometry. Volcano plot showing differentially expressed proteins. Proteins regulated with *P* < 0.05 are depicted in red. **B,** Rowwise Z-score heat maps showing upregulated and downregulated proteins in BCSC5 culture cells or xenograft-derived tumors from two or three biological replicates (BR) as indicated. **C,** Migration of γδ T cells (CD3^+^γδTCR^+^) in response to BCSC5 culture cells and xenograft-derived tumor cells was determined in a transwell assay. Basal migration towards medium was set to 1.0 and fold changes from three independent experiments using the same 3 healthy donors in each experiment were pooled and analyzed using one-sample Wilcoxon test. **D,** Migration of Vδ1^+^ (CD3^+^γδTCR^+^Vδ1^+^) and Vδ2^+^(CD3^+^γδTCR^+^Vδ2^+^) T cells in response to BCSC5 culture cells and xenograft-derived tumor cells was determined in a transwell assay. Basal migration toward medium was set to 1.0 and fold changes from three independent experiments using the same 3 healthy donors in each experiment were pooled. One-sample *t* test (for Vδ1^+^) or one-sample Wilcoxon test (for Vδ2^+^). *, *P* ≤ 0.05; **, *P* ≤ 0.01; ****, *P* ≤ 0.0001. Non-adj., non-adjusted.

To discover proteins and mechanisms involved in the xenograft immune escape *in vivo*, we utilized LC/MS-MS to compare the xenograft-derived tumor cell proteomes of all treatment groups with the proteome of BCSC5 culture cells. These analyses revealed that tumor growth in NOD SCID mice drastically changed the proteome of the cells. Around 5,600 proteins were differentially expressed by vehicle-treated xenograft cells compared with BCSC5 culture cells (Fig. 5A). Next, we more closely examined the chemokine secretion profiles of xenografted BCSC5 compared with culture cells to exclude the possibility that xenograft-derived cells lost their ability to attract γδ T cells to the tumor site *in vivo.* Of the detected chemokines, CXCL5 expression was reduced in the xenograft-derived tumor cells compared with BCSC5 culture cells, while CCL20 and CCL28 expression was augmented (Fig. 5B). The rest of the detected chemokines remained unchanged (CXCL12, CXCL14, and CXCL16). None of the observed changes could be associated with a specific treatment. Next, we analyzed γδ T-cell migration toward BCSC5 culture cells and freshly isolated xenograft-derived tumor cells. Indeed, cells derived from the xenograft attracted γδ T cells more efficiently than BCSC5 culture cells (Fig. 5C). We assayed Vδ1^+^ and Vδ2^+^ T cells separately and observed that Vδ2^+^ T cells migrated more efficiently toward cells derived from the xenograft compared with Vδ1^+^ T cells (Fig. 5D). These results highlight that xenografts escaped γδ T-cell immunotherapy by other means than reducing γδ T-cell attraction.

### BCSC5 cells differentiate *in vivo* and downregulate the expression of **γδ** T-cell ligands

**Figure 6.**
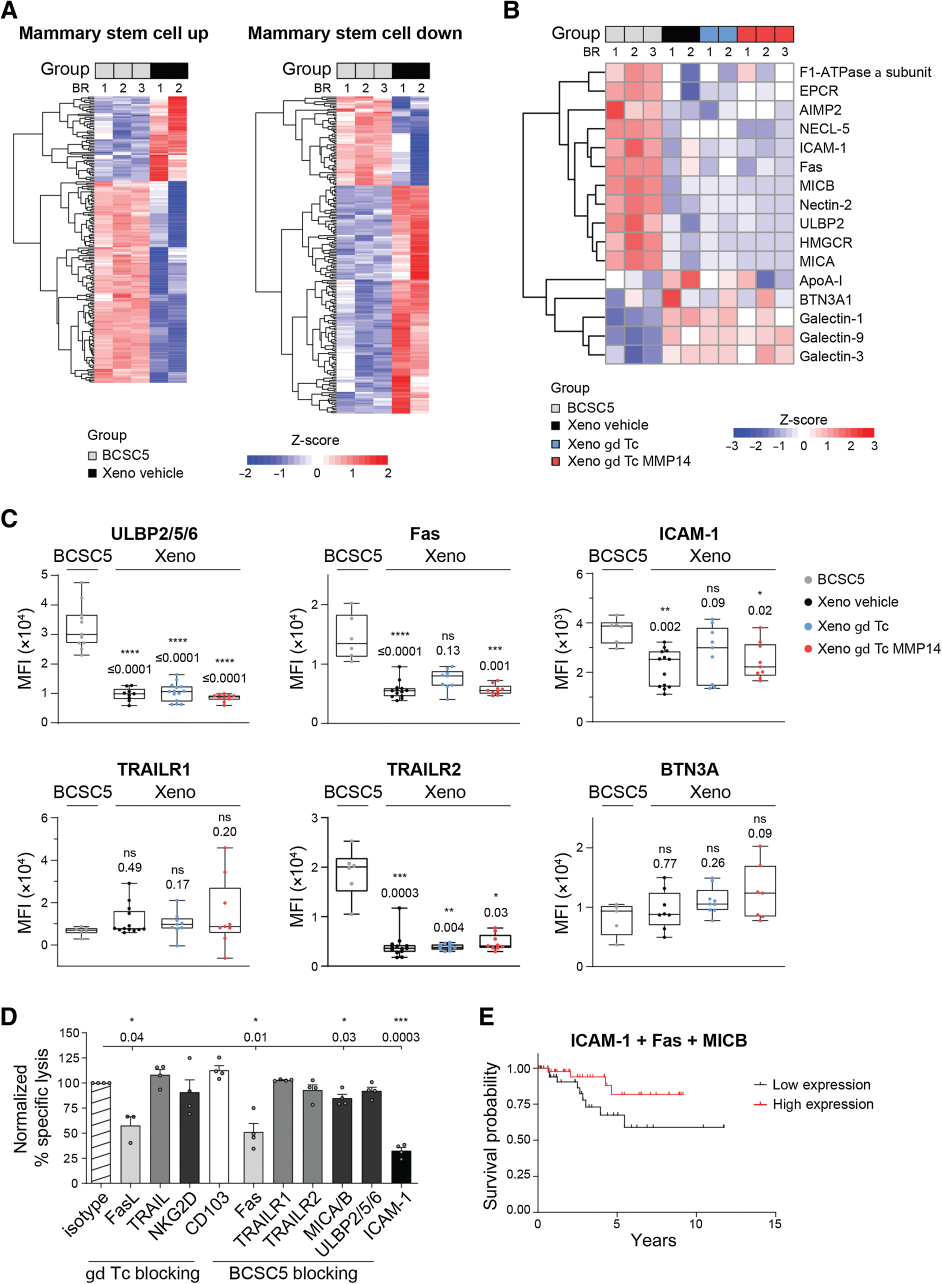
BCSCs differentiate *in vivo* and downregulate the expression of proteins recognized by γδ T cells. **A,** Rowwise Z-score heat maps showing upregulated and downregulated proteins in BCSC5 culture cells or xenograft-derived tumors from two or three biological replicates as indicated (BR). **B,** Rowwise Z-score heat map for proteins involved in target cell recognition by γδ T cells or in immune response modulation. **C,** Flow cytometry-based analysis of BCSC5 culture cells and xenograft-derived tumor cells. MFI of at least five tumors per group is shown (median, minimum to maximum). One-way ANOVA followed by Dunnett *post hoc* test comparing xenograft-derived cells with culture cells (for ULBP2/5/6, ICAM-1, and BTN3A). Kruskal-Wallis test followed by Dunn *post hoc* test comparing xenograft-derived cells with culture cells (for Fas, TRAILR1, and TRAILR2). **D,** Killing of BCSC5 by γδ T cells requires multiple ligand–receptors interactions. *In vitro* killing of luciferase-expressing BCSC5 cells by γδ T cells. γδ T cells or BCSC5 culture cells were pretreated with the indicated blocking antibodies for 1 hour. Cells were cocultured at an E:T ratio of 10:1 for 8 hours in the presence of the blocking antibodies or the corresponding isotype controls. Corresponding receptor-ligand pairs are represented in the same color. Isotype antibody control was set to 100% for normalization in each experiment. Results for 3 to 4 healthy donors of γδ T cells from three independent experiments were pooled (means ± SEM). One-sample *t* test against hypothetical value of 100. **E,** Cox regression of progression-free survival for patients with TNBC sorted by high (top quartile) and low (bottom quartile) average clustered expression of the Fas, MICB, and ICAM-1. β Cox coefficient was −0.33 and the Cox *P* value was 0.07 suggesting that high expression of the three proteins correlates with a better survival prognosis. *, *P* ≤ 0.05; **, *P* ≤ 0.01; ***, *P* ≤ 0.001; ****, *P* ≤ 0.0001.

Next, we performed pathway analyses on our proteomic data and verified that BCSC5 culture cells exhibited a mammary stem cell (MSC) signature. However, proteins usually expressed by MSCs were significantly downregulated in xenograft-derived tumor cells and, vice versa, proteins usually lowly expressed in MSCs were upregulated (Fig. 6A). These findings indicated that BCSC5 cells differentiated *in vivo* losing their breast stem cell signature, despite BCSC-derived cells preserving the patient's original molecular tumor subtype (22, 34).

In addition to changes affecting stemness, the transfer and growth of BCSC5 *in vivo* drastically changed the expression levels of well-known γδ T-cell ligands and other molecules involved in cancer cell recognition by γδ T cells (Fig. 6B). We found that a variety of these proteins were specifically downregulated in xenograft-derived tumor cells and that these changes were independent of the γδ T-cell treatment (Fig. 6B). We validated the reduced expression of ULBP-2/5/6, Fas, and ICAM-1 via flow cytometry (Fig. 6C). All these proteins have been previously described to be involved in tumor cell killing by γδ T cells (13, 36–38). Although TRAILR1 and TRAILR2 were not identified in our proteomic study, TRAIL–TRAILR interactions induce γδ T cell–mediated killing (39). While TRAILR1 expression was not significantly changed on xenograft-derived tumor cells compared with BCSC5 culture cells, TRAILR2 levels were drastically reduced (Fig. 6C). Taken together, the loss of the above-mentioned proteins might explain why γδ T cells cannot efficiently recognize and kill xenograft-derived tumor cells. We next analyzed TCGA (https://www.cancer.gov/tcga) datasets for patients with TNBC scoring for the expression level of Fas, MICA/B, TRAILR1/2, and ICAM-1 individually. Cox proportional hazards analysis failed to reveal significant prolonged survival for patients with high expression of each of the analyzed proteins (Supplementary Fig. S5). This analysis suggests that each of these proteins individually cannot predict suitability for eventual immunotherapies using γδ T cells.

Next, we aimed to elucidate the mechanisms involved in the killing of BCSC5 by γδ T cells to narrow down which phenotypic changes might be mainly responsible for the immune escape of xenografted BCSC5. We investigated the role of Fas, MICB, TRAILR1/2, and ICAM-1 in the recognition and subsequent killing of BCSC5 by performing cytotoxicity experiments using blocking antibodies. We found that γδ T cell–mediated killing of BCSC5 involves Fas-FasL interactions, MICA/B engagement and, most drastically, ICAM-1 binding (Fig. 6D). In contrast, TRAIL-TRAILR interactions, ULBP2/5/6 engagement, and the binding of CD103 (integrin α E) did not play a major role in BCSC5 killing (Fig. 6D). These results indicate that among the phenotypic changes observed upon xenotransplantation, the loss of Fas, MICA/B and ICAM-1 expression might play a crucial role in protecting these cells from γδ T cell–mediated cytotoxicity. Our data also suggest that γδ T cells might require multiple receptor–ligand interactions to efficiently kill BCSCs. We hence clustered TCGA data from patients with TNBC based on the level of expression of the three key proteins Fas, MICB and ICAM-1. Cox proportional hazards analysis showed that patients expressing high levels of these proteins have a 1.4 times lower 5-year mortality risk (Fig. 6E).

### Zoledronate sensitizes xenograft-derived tumor cells to **γδ** T cell–mediated killing

**Figure 7.**
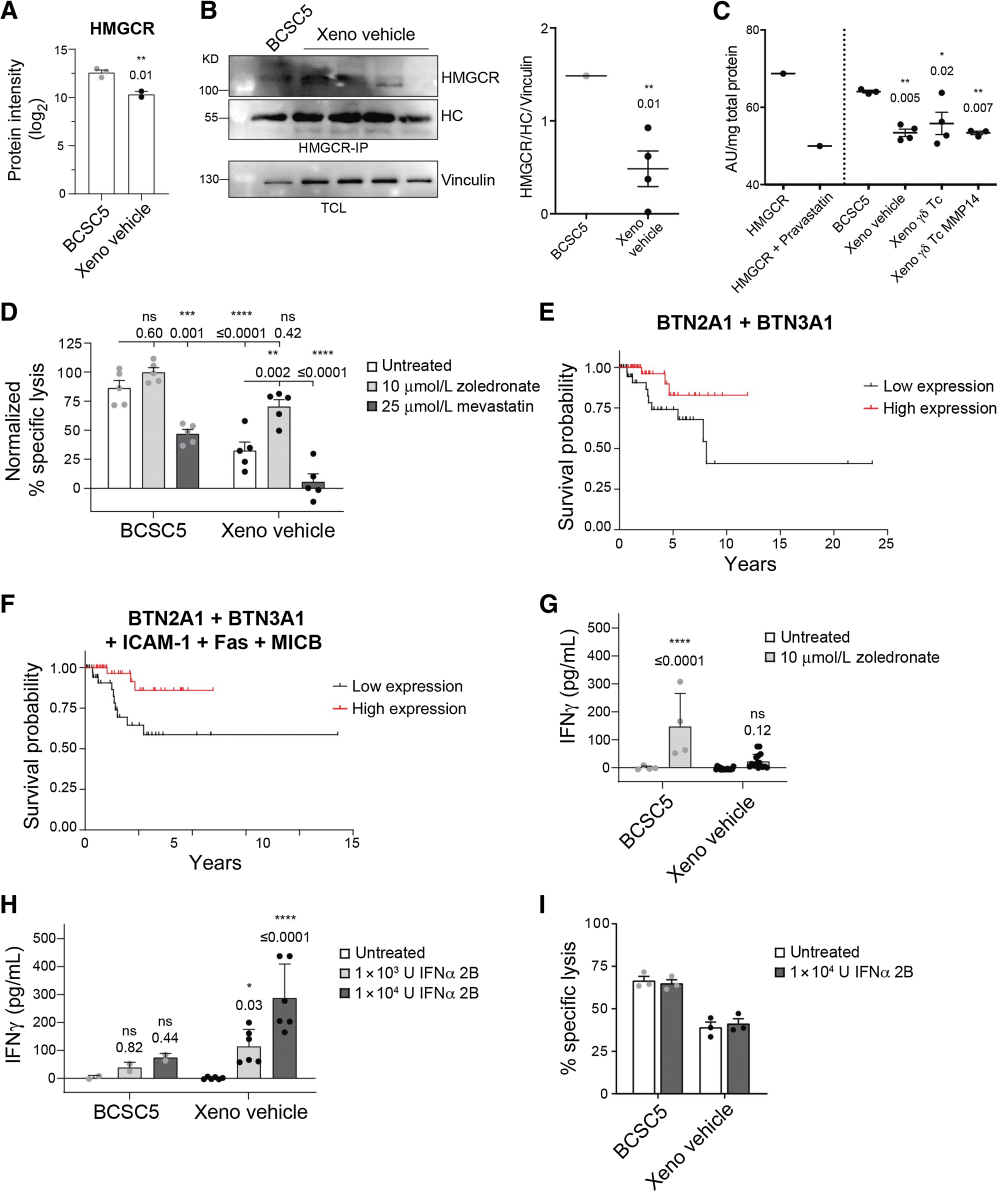
Zoledronate sensitizes xenograft-derived tumor cells to γδ T cell–mediated killing. **A,** HMGCR expression levels determined by mass spectrometry. Protein intensities (log_2_) are shown as means ± SEM for culture and xenograft-derived tumor cells (vehicle). Unpaired *t* test, two tailed. **B,** HMGCR expression levels determined by IP and Western blot analysis. Quantification shows mean ± SEM for xenograft-derived tumor cells (vehicle). Unpaired *t* test, two tailed. **C,** HMGCR activity on total cellular lysates, purified HMGCR is used as positive control and purified HMGCR incubated with pravastatin, as negative control. Means ± SEM are shown. One-way ANOVA followed by Dunnett *post hoc* test. **D,***In vitro* killing of ^51^Cr-labeled tumor cells by (MACS)-separated Vδ2^+^ T cells. Target cells were pretreated with 10 μmol/L zoledronate or 25 μmol/L mevastatin for 2 hours. Cells were cocultured at an E:T ratio of 10:1 for 20 hours in the presence of the indicated drug. Results for 3 healthy donors of γδ T cells from four independent experiments were pooled (means ± SEM). Killing of BCSC5 culture cells in the presence of zoledronate was set to 100% for normalization. Two-way ANOVA followed by Tukey *post hoc* test comparing all groups with each other. **E,** Cox regression of progression-free survival for patients with TNBC sorted by high (top quartile) and low (bottom quartile) average expression of both proteins BTN2A1 and BTN3A1. β Cox coefficient was −0.8 and the Cox *P* value was 0.008. **F,** Cox regression of progression-free survival for patients sorted by high (top quartile) and low (bottom quartile) average clustered expression of BTN2A1, BTN3A1, Fas, MICB, and ICAM-1. β Cox coefficient was −0.6 and the Cox *P* value was 0.02. **G,** IFNγ secretion by (MACS)-separated Vδ2^+^ T cells in response to BCSC5 culture cells or xenograft-derived tumor cells was determined. Target cells were pretreated with 20 μmol/L zoledronate for 2 hours before coculture with γδ T cells at a ratio of 1:1 in the presence of 10 μmol/L zoledronate. The culture supernatant after 24 hours was analyzed for secreted IFNγ using ELISA. Results were baseline corrected by the corresponding amounts of IFNγ secreted by γδ T cells alone. Results for 3 healthy donors of γδ T cells from two experiments testing eight individual tumors were pooled (means ± SEM). Two-way ANOVA followed by Sidak *post hoc* test comparing treatment with respective untreated groups. **H,** IFNγ secretion by (MACS)-separated Vδ2^+^ T cells in response to BCSC5 culture cells or xenograft-derived tumor cells was determined. Tumor cells were pretreated with 10^3^ U or 10^4^ U IFNα2B for 1 hour before coculture with γδ T cells at a ratio of 1:1 in the presence of IFNα2B. The culture supernatant after 24 hours was analyzed for secreted IFNγ using ELISA. Results were baseline corrected by the corresponding amounts of IFNγ secreted by γδ T cells alone. Results for 2 healthy donors of γδ T cells from one experiment testing three individual tumors were pooled (means ± SEM). Two-way ANOVA followed by Dunnett *post hoc* test comparing treatment to respective untreated groups. **I,***In vitro* killing of ^51^Cr-labeled tumor cells by (MACS)-separated Vδ2^+^ T cells. Target cells were pretreated with 10^4^ U IFNα2B for 1 hour and then cocultured with γδ T cells in the presence of IFNα2B at an E:T ratio of 10:1 for 20 hours. Representative results for one healthy donor of γδ T cells is shown. Indicated are technical replicates (means ± SEM). *, *P* ≤ 0.05; **, *P* ≤ 0.01; ***, *P* ≤ 0.001; ****, *P* ≤ 0.0001.

In addition to proteins directly involved in killing, we identified HMGCR to be significantly downregulated in the xenograft-derived tumor cells (Fig. 6B; Fig. 7A and B). The SREBP-SCAP complex regulates the transcription of *HMGCR*. Indeed, SREBP-SCAP protein levels were significantly reduced in xenograft-derived tumor cells (Supplementary Fig. S6). Consequently, *HMGCR* transcripts were reduced both in the xenograft-derived tumor cells as well as in the original patient tumors indicating that *in vivo* differentiation of BCSCs reduces *HMGCR* both in mouse models and in patients (Supplementary Fig. S6). Furthermore, HMGCR activity was significantly reduced in lysates obtained from xenograft-derived tumor cells (Fig. 7C). HMGCR is the rate-limiting enzyme of the mevalonate pathway, which regulates the levels of pAgs in tumor cells (40). These pAgs can be recognized by the Vγ9Vδ2 TCR expressed by Vδ2^+^ T cells in the context of butyrophilin 3A1 (BTN3A1) and 2A1 (BTN2A1; refs. 41, 42). Expression of BTN3A1, however, was not significantly altered in xenograft-derived tumor cells, and BTN2A1 was not detected in the proteomic study (Fig. 6B). Likewise, no changes were observed using an antibody recognizing all BTN3A isoforms (Fig. 6C). Considering that the BTN3A1 levels were comparable between BCSC5 culture cells and xenograft-derived tumor cells, we hypothesized that the loss of HMGCR expression was responsible for unresponsiveness of γδ T cells due to low pAg levels in the tumor cells. To further explore this, we performed cytotoxicity assays in the presence of zoledronate or mevastatin, two well-studied drugs interfering with the mevalonate pathway and modulating pAg levels (43, 44). Zoledronate, most probably via the accumulation of pAgs, increased γδ T-cell cytotoxicity toward xenograft-derived tumor cells (Fig. 7D). The cytotoxicity in the presence of zoledronate was similar to the killing of untreated BCSC5 culture cells (Fig. 7D). In contrast, the accumulation of pAgs by zoledronate in BCSC5 culture cells failed to increase γδ T-cell cytotoxicity toward these cells, suggesting that this recognition axis was already saturated (Fig. 7D). Reducing pAg levels by mevastatin led to reduced killing of BCSC5 culture cells and almost completely abolished the killing of xenograft-derived cells (Fig. 7D). These findings show that the killing of xenograft-derived tumor cells by γδ T cells almost exclusively relied on the recognition of pAgs and that these cells can be sensitized to γδ T cell–mediated killing by zoledronate treatment. Both BTN2A1 and BTN3A1 are necessary for the recognition of pAgs by γδ T cells (41, 42). The correlation between the mRNA levels of *BTN2A1* and *BTN3A1* does not change between mammary healthy tissue (0.5988) and TNBC patient-derived samples (0.5924). We clustered patients with TCGA TNBC by the expression levels of both BTN2A1 and BTN3A1 and found that high expression of both proteins significantly correlated with increased survival. Patients with high levels of BTN2A1 and BTN3A1 mRNA had a two times lower 10-year mortality risk (Fig. 7E). BCSC5 culture cells, in contrast, can still be partially recognized in the presence of mevastatin, in line with the finding that BCSC5 can be killed by several mechanisms, namely those involving Fas, MICB, and ICAM-1 as detailed above (Figs. 7D and 6D). Thus, we clustered patients with TCGA TNBC by the mRNA levels of BTN2A1 and BTN3A1, which are key for the recognition of differentiated cells, and Fas, MICB, and ICAM-1, which seem to be central for the recognition and killing of BCSCs. Patients with high levels of these proteins showed significantly increased survival having a 1.4 times lower 5-year mortality risk (Fig. 7F). Gene set enrichment analysis of TCGA data revealed that patients with TNBC clustered by high (top quartile) average expression of BTN2A1, BTN3A1, Fas, MICB, and ICAM-1 exhibited higher expression of BCSC genes and gene signatures associated to immune response, inflammation, IFNγ and IFNα responses, cytokine and chemokine signaling and immune-mediated cytotoxicity (Supplementary Fig. S7) suggesting a favorable tumor immune microenvironment. To deepen into this observation, we applied the “deep deconvolution” CIBERSORT algorithm to TCGA data to deduce the immune cell composition (45) and compared the patients with TNBC with high *versus* low average expression of BTN2A1, BTN3A1, Fas, MICB, and ICAM-1 (Supplementary Fig. S8). CD4^+^ memory T cells, resting and activated, CD8^+^ T cells, and antitumor M1 macrophages were enriched in patients with TNBC with high average expression. In contrast, M2 tumor-promoting macrophages were significantly reduced. The assessment of tumor-infiltrating human γδ T cells by deconvolution has proven to be challenging, therefore we analyzed genes specifically upregulated in human γδ T cells that have been identified by machine learning approaches (46). Indeed, these human γδ T cell–specific genes were clearly upregulated in those patients with TNBC clustered by high (top quartile) average expression of BTN2A1, BTN3A1, Fas, MICB, and ICAM-1 (Supplementary Fig. S8). Thus, expression of these proteins might be an instrumental tool to identify patients with TNBC suitable for γδ T cell–based immunotherapy approaches.

In addition to cytotoxicity, γδ T cells play a critical role in protective immune responses against tumor development by providing an early source of the proinflammatory cytokine IFNγ (16, 47). IFNγ plays a manifold role in activating anticancer immunity. For instance, IFNγ promotes the activity of tumor-triggered αβ T cells and inhibits the differentiation and activation of regulatory αβ T cells (48). The current view is that IFNγ-producing cells are endowed with potent cytotoxic functions during antitumor responses (49). In our settings, γδ T cells, with or without zoledronate treatment, did not respond with IFNγ secretion to xenograft-derived tumor cells (Fig. 7G), which might limit the protective immune response against tumor development. It has been previously shown that IFNα can mediate an increase in IFNγ secretion by pAg-activated Vδ2^+^ T cells (50). In line with this report, the pretreatment of tumor cells with IFNα induced γδ T cell–mediated IFNγ secretion in response to xenograft-derived tumor cells (Fig. 7H) while it failed to rescue γδ T cell–mediated cytotoxicity toward xenograft-derived tumor cells (Fig. 7I). Taken together, the treatment with IFNα  facilitated the activation of γδ T cells to produce IFNγ, which might be therapeutically interesting in clinical settings in which tumor-triggered αβ T cells play an important role. Yet, this boost of γδ T cells to produce IFNγ  did not translate into increased cytotoxicity toward BCSCs highlighting that a distinct signals and/or a different threshold of activation are needed to produce IFNγ or to induce killing by γδ T cells. The molecular identification and targeting of the pathways regulating these two effector functions are beyond the scope of the current study.

In summary, our proteomic studies and biological validations strongly support the hypothesis that BCSC5 culture cells differentiated *in vivo*. This differentiation involved the upregulation of inhibitory T-cell receptors, the loss of stem cell characteristics, the reduction of numerous γδ T-cell ligands and a decrease in pAg levels, in sum preventing efficient recognition and killing by γδ T cells.

## Discussion

It has been suggested that BCSCs are responsible for therapy resistance and metastatic dissemination in breast cancer, which is the leading cause of cancer deaths among women worldwide. Until now, treatment-resistant BCSCs have only been poorly characterized, and targeted therapeutics have yet to be identified. We have recently established an optimized culture system to expand human BCSCs that faithfully reproduce the original patient's tumor characteristics and are therefore an ideal cellular platform to test novel therapeutics (22, 34). Here, we aimed to investigate the susceptibility of these BCSCs to immunotherapy using primary human γδ T cells. We showed that patient-derived triple-negative BCSCs are targeted by both Vδ1^+^ and Vδ2^+^ primary expanded γδ T cells. However, orthotopically xenografted BCSC5, the BCSC line best recognized by γδ T cells in our study, was refractory to γδ T-cell immunotherapy. We demonstrated that attraction and/or migration toward xenografted cells was not the reason for this unexpected *in vivo* outcome. Both Vδ1^+^ and Vδ2^+^ T cells migrated efficiently toward BCSC CM and this migration was even increased toward xenografted cells. The chemokine receptors expressed on the expanded γδ T cells were compatible with the secretion profile of xenografted cells.

Solid tumors, including breast cancer, are often surrounded by a dense ECM preventing the efficient infiltration by immune cells (25). A high stromal content has been associated with poor prognosis in TNBC, and an accumulation of γδ T cells in the tumor stroma contributes to this observation (51). Detailed analyses of γδ T-cell migration with respect to the tumor stroma are not yet available. Here, we found that exogenously expressing MMP14 conferred promigratory functions to primary γδ T cells in a 3D model for basement membranes. Crossing of the basement membrane is of critical relevance for γδ T cells to extravasate from the blood stream at the tumor site (52). Using viable slices of BCSC5-derived xenograft tumors, we showed that  γδ T-cell migration was accelerated inside of tumor islets compared with the ECM-rich tumor stroma similar to previous observations made for αβ T cells (19). MMP14 expression increased γδ T-cell migration speed exclusively in the tumor stroma and not inside the tumor tissue, supporting the functional role of MMP14 in cleaving the peritumoral ECM (31). These findings are in line with a previous report showing that expressing the secreted ECM-modifying enzyme heparanase in human CAR αβ T cells promoted tumor rejection in melanoma and neuroblastoma xenograft models (21). In another study, inhibition of the ECM cross-linking enzyme lysyl oxidase improved antitumor responses in combination with checkpoint inhibition due to increased αβ T-cell migration and infiltration (53). We hypothesize that the overexpression of MMP14 might have certain advantages over the expression of secreted heparanase or the systemic application of lysyl oxidase inhibitors, as MMP14 is a membrane-anchored protein lowering the risk of structural changes outside of the tumor tissue. Although we have not observed any side effects related to MMP14 expression in mice receiving γδ T-cell treatment, the application of MMP14 will have to be strictly monitored with respect to biodistribution and its impact on healthy tissues in future studies.

Despite increased chemoattraction and MMP14-mediated peritumoral migration, γδ T cells failed to control BCSC5-derived tumors *in vivo*. Remarkably, xenografted cells showed reduced capacity to activate γδ T cells and thereby reduced susceptibility to γδ T cell-mediated cytotoxicity. Despite our BCSCs reliably reproducing many original patient's tumor characteristics (22, 34), they underwent major changes in their proteomic signature after xenograft and growth *in vivo*. BCSC5 cells lost their stem cell characteristics and downregulated a plethora of surface proteins key for immunosurveillance by γδ T cells after transplantation and growth *in vivo.* Intriguingly, this *in vivo* differentiation was not a consequence of mechanisms induced by the immune system or the immunotherapy, because it was equally observed in immunodeficient mice with or without the presence of primary human γδ T cells. Xenografted cells exhibited increased surface expression of the inhibitory T-cell ligands PD-L1 and PD-L2 compared with parental BCSC5 cells, in line with studies associating TNBC with high expression levels of PD-L1 (54). This increased expression of inhibitory T-cell ligands might explain the reduced susceptibility to be killed by γδ T cells. However, a combinatorial treatment of xenografts with γδ T cells and anti–PD-1 did not result in reduced xenograft growth. Similarly, Li and colleagues have described the inefficacy of this treatment regimen against TNBC MDA-MB-231-derived xenografts (54).

Because increased PD-L1 levels were not responsible for the immune evasion of xenografted cells, we searched for additional molecules involved in BCSC recognition and compared their expression levels before and after *in vivo* growth. Our results revealed that recognition and killing of BCSC5 by γδ T cells required the engagement of multiple receptor–ligand pairs. Killing was thus dependent on ICAM-1 binding, MICB, pAgs/BTN2A1/BTN3A1 and on Fas–FasL interactions. Indeed, clustering patients with TNBC for high expression of these proteins significantly improved survival prognosis for this group. In αβ T cells, ICAM-1 is key for the formation of a functional immune synapse and to enable CAR αβ T-cell entry into solid tumors (55). Low ICAM-1 expression levels on breast cancer cells made them resistant to αβ T-cell killing (16). Likewise, human pancreatic cancer cell lines lacking ICAM-1 were poorly bound and killed by γδ T cells *in vitro* (36). Thus, our results support that ICAM-1–mediated intercellular interactions facilitate γδ T cell–mediated recognition and killing of BCSCs by Fas–FasL interactions and pAgs. However, xenograft-derived tumor cells lost Fas, MICB, and ICAM-1 expression, and downmodulated HMGCR, the rate-limiting enzyme of the mevalonate pathway producing pAgs. These changes are most probably responsible for the escape of xenograft-derived tumor cells from γδ T-cell recognition. Yet we observed some residual cytotoxicity toward xenograft-derived tumor cells that was exclusively dependent on pAg recognition as pharmacologic inhibition of HMGCR by mevastatin abolished cytotoxicity. Accumulation of pAgs by zoledronate pretreatment overcame the resistance of xenograft-derived tumor cells to γδ T cell–mediated killing. The clinical application of zoledronate is FDA approved for the treatment of osteoporosis and bone metastasis. Therefore, combinatorial therapy approaches using zoledronate and the adoptive transfer of γδ T cells represent a promising option to simultaneously tackle BCSCs and their differentiated progeny.

Our observation that BCSCs can be better recognized by γδ T cells than their differentiated progeny apparently opposes previous reports using the expression of CD44 and CD24 to define stemlike cells (CD44^hi^CD24^lo^) and their non–stem cell counterparts (CD44^hi^CD24^hi^). Sorted stemlike cells from the triple-negative SUM149 cell line and from PDX401 cells were less efficiently killed by γδ T cells compared with non–stem cell counterparts (17). This study identified MICA shedding from the tumor cell surface as a mechanism to escape γδ T-cell recognition. MICA surface levels were unchanged between the differentiated xenograft cells and the BCSCs suggesting that MICA shedding does not play a major role in the escape of BCSC5-derived xenografts *in vivo*. To the best of our knowledge, only one other study investigated the response of γδ T cells against BCSCs and nonstem cells derived from *ras*-transformed human mammary epithelial cells, and found that both cell populations were equally resistant to γδ T cell–mediated killing (16). Yet, both populations could be sensitized by zoledronate treatment in line with our results. Taken together, these discrepancies among studies underline the importance of perceiving immunotherapeutic approaches as individualized medicine, which might have to be tailored for each patient.

In addition, our results highlight a previously unnoticed dichotomy, namely that IFNγ responses and cytotoxicity by γδ T cells do not necessarily correlate. Zoledronate enhanced cytotoxicity by γδ T cells but failed to promote IFNγ secretion. In contrast, IFNα treatment increased γδ T cell–mediated IFNγ secretion but failed to enhance γδ T cell-mediated cytotoxicity. This result suggests that IFNα might represent an attractive tool to be used in combinatorial therapies to induce synergistic effects of γδ and αβ T-cell responses.

In summary, our data show that patient-derived triple negative BCSCs are targetable by expanded γδ T cells. However, *in vivo* growth of these BCSCs leads to their differentiation into cells that lost stemness and ligands to activate γδ T-cell responses and thereby, escaped from efficient killing by γδ T cells. Still, γδ T cells residually killed *in vivo* differentiated cells by recognizing pAgs. This killing could be increased to the level of BCSC5 killing by zoledronate. In all, we propose that a combinatorial therapy using γδ T cells and zoledronate represents a valuable approach to target triple-negative BCSCs and non–stem cells alike. Furthermore, IFNα treatment could induce IFNγ production by γδ T cells, and thereby induce a first source of IFNγ promoting ICAM-1 expression, T-cell entry into solid tumors (55) and further endogenous αβ T-cell responses in immunocompetent settings.

## Supplementary Material

Supplementary Video S1 UT Supplementary Video S1 UT

Supplementary Video S2 MMP14 Supplementary Video S2 MMP14

Supplementary Figures and Figure Legends Supplementary Figures and Figure Legends

Supplementary Table S1 Supplementary Table S1
