# Supplementary material for: Breast cancer stem cell-derived tumors escape from γδ T-cell immunosurveillance in vivo by modulating γδ T-cell ligands
Source: Cancer Immunol Res. Author manuscript; Available in PMC 2023 Jun 3. (PMC10236150; doi:10.1158/2326-6066.CIR-22-0296)
Supplement: Supplementary Data [file EMS173179-supplement-Supplementary_Data.docx]

**Supplementary Data**

**Breast cancer stem cell-derived tumors escape from γδ T cell immunosurveillance *in vivo* by modulating γδ T cell ligands**

Katrin Raute^1 2 3 4^, Juliane Strietz^1 2 3 #^, Maria Alejandra Parigiani^1 2 3 #^, Geoffroy Andrieux^5,6^, Oliver S. Thomas^1 2 4^, Klaus M. Kistner^1 2 3^, Marina Zintchenko^1 2 3^, Peter Aichele^3^, Maike Hofmann^7^, Houjiang Zhou^8^, Wilfried Weber^1 2^ , Melanie Boerries^5,6^, Mahima Swamy^9^, Jochen Maurer^10^ and Susana Minguet^1 2 3 *^

1. Faculty of Biology, University of Freiburg, Freiburg, Germany.
2. Signalling Research Centres BIOSS and CIBSS, University of Freiburg, Freiburg, Germany.
3. Center of Chronic Immunodeficiency CCI and Institute for Immunodeficiency, University Clinics and Medical Faculty, Freiburg, Germany.
4. Spemann Graduate School of Biology and Medicine (SGBM), University of Freiburg, Freiburg, Germany.
5. Institute of Medical Bioinformatics and Systems Medicine, Medical Center - University of Freiburg, Faculty of Medicine, University of Freiburg, Freiburg, Germany.
6. German Cancer Consortium (DKTK) Partner Site Freiburg, German Cancer Research Center (DKFZ), Heidelberg, Germany
7. Department of Medicine II (Gastroenterology, Hepatology, Endocrinology and Infectious Diseases), Freiburg University Medical Center, Faculty of Medicine, University of Freiburg, Freiburg, Germany
8. Medical Research Council Protein Phosphorylation and Ubiquitylation Unit, University of Dundee, Dundee, United Kingdom.
9. Cell Signalling and Immunology, University of Dundee, Dundee, United Kingdom.
10. Department of Obstetrics and Gynecology, University Hospital Aachen (UKA), Aachen, Germany.

- Corresponding author: [susana.minguet@biologie.uni-freiburg.de](mailto:susana.minguet@biologie.uni-freiburg.de)
- ^#^ Authors contributed equally
- The authors declare no potential conflicts of interest


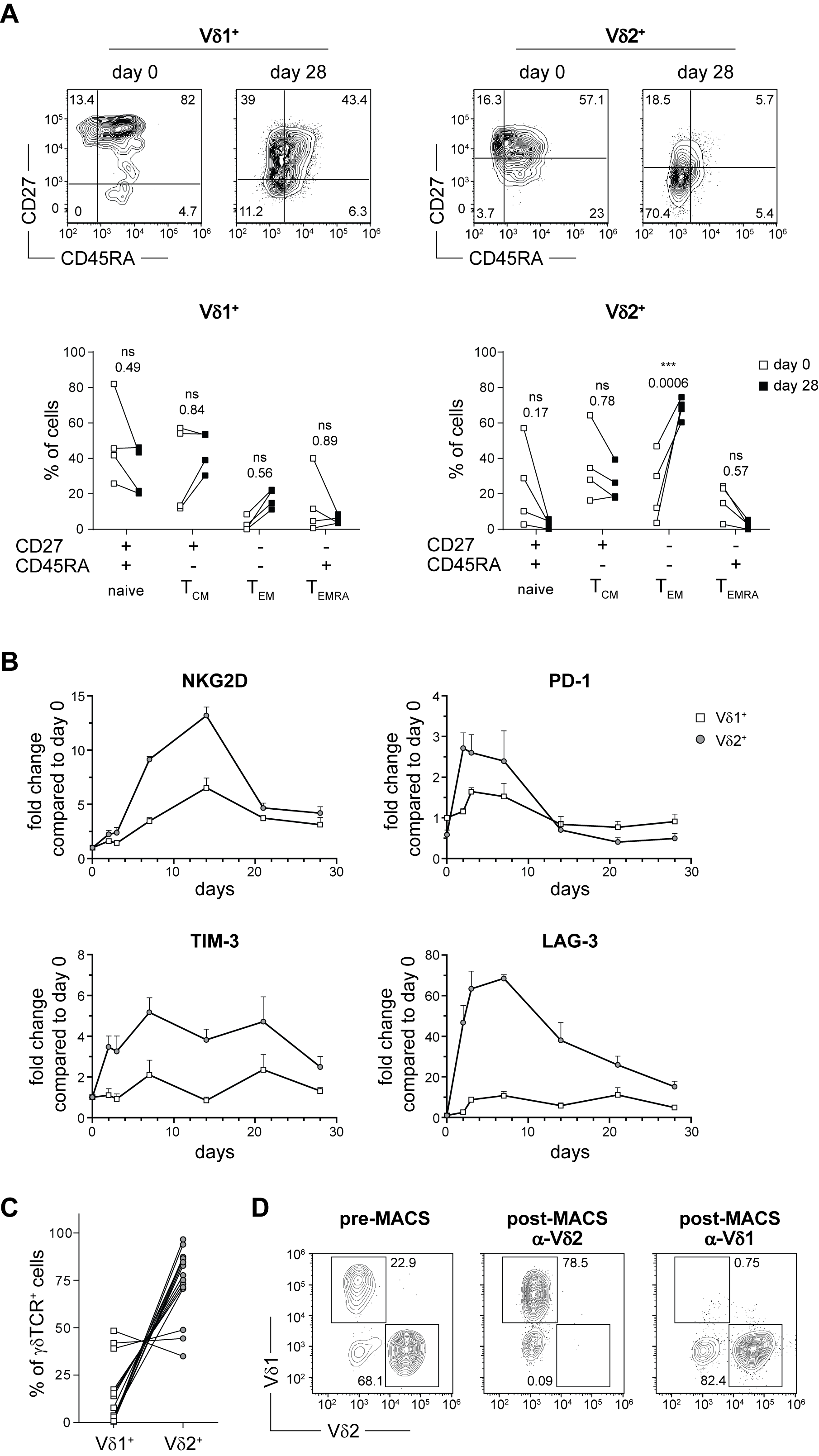


**Supplementary Fig. S1: Expanded human γδ T cells are mainly of the central memory and effector memory phenotype.**

**(A)** Flow cytometry‑based analysis of Vδ1^+^ and Vδ2^+^ T cells on day 0 and day 28 after peripheral blood mononuclear cell (PBMC) stimulation with 10 µg/ml Concanavalin A (ConA), 10 ng/ml IL-2 and 10 ng/ml IL-4. Cells were stained and gated to distinguish Vδ1^+^ and Vδ2^+^ T cells and phenotyped for the expression of CD27 and CD45RA: naïve T cell, CD27^+^CD45RA^+^; central memory T cell (T_CM_), CD27^+^CD45RA^‑^; effector memory T cell (T_EM_), CD27^-^CD45RA^-^; CD45RA^+^ effector memory T cell (T_EMRA_), CD27^-^CD45RA^+^. Gates were based on fluorescence-minus-one (fmo) controls. Shown are representative dot plots from one healthy donor (upper panels) and statistical analysis of pooled results of four healthy donors stained simultaneously (lower panels). Two-way ANOVA followed by Sidak’s post hoc test comparing stimulated or co-cultured cells to the corresponding medium control. **(B)** PBMCs stimulated as in (A) were stained and gated to distinguish Vδ1^+^ and Vδ2^+^ T cells and the expression of NKG2D, PD-1, TIM-3 and LAG-3 was monitored over time. Data were normalized to the mean fluorescence intensity (MFI) at day 0. Pooled data of three healthy donors from one experiment are shown (means ± SEM). **(C)** In vitro killing of luciferase-expressing BCSC5 cells was assayed after 18 h of co-incubation at an effector to target (E:T) ratio of 10:1 at the indicated times after expansion start (days). Results from three healthy donors (HD) are shown (means ± SEM). Each dot (circle, triangle or square) represents a healthy donor. **(D)** Analysis of the percentage of Vδ1^+^and Vδ2^+^ T cells among γδTCR^+^ gated cells. Shown are the results for 15 independent expansion cultures from different donors. **(E)** Representative flow cytometry dot plots of γδ T cell cultures before and after magnetic activated cell sorting (MACS). Vδ2^+^ or Vδ1^+^T cells were depleted from cultures by using α-Vδ2 or α-Vδ1 antibodies, respectively. Shown are γδTCR^+^ gated cells. *** p≤0.001.


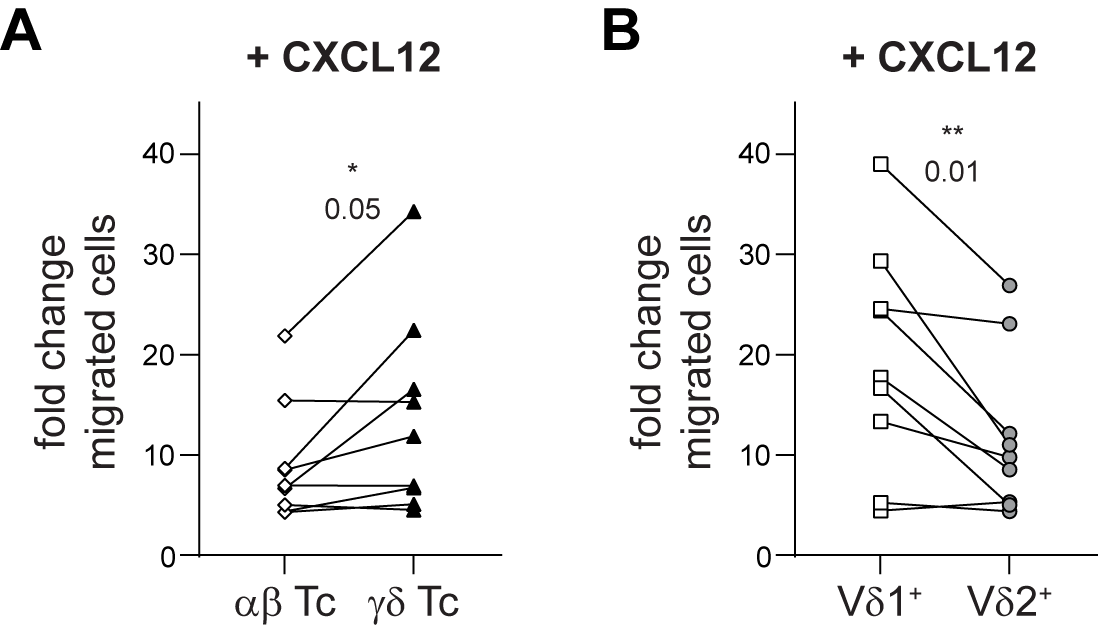


**Supplementary Fig. S2: T cell migration towards the chemokine CXCL12.**

**(A)** Migration of αβ T cells (CD3^+^γδTCR^-^) and γδ T cells (CD3^+^γδTCR^+^) in response to CXCL12 (50 ng/ml) was determined in a transwell assay as described in Fig. 2 (a). Basal migration towards medium was set to 1.0 and fold changes from three independent experiments using the same three healthy donors in each experiment were pooled. Wilcoxon signed-rank test. **(B)** Migration of Vδ1^+^ (CD3^+^γδTCR^+^Vδ1^+^) and Vδ2^+^ (CD3^+^γδTCR^+^Vδ2^+^) T cells in response to CXCL12 (50 ng/ml) was determined in a transwell assay, which was analyzed as detailed in (A). Wilcoxon signed-rank test.* p≤0.05, ** p≤0.01.


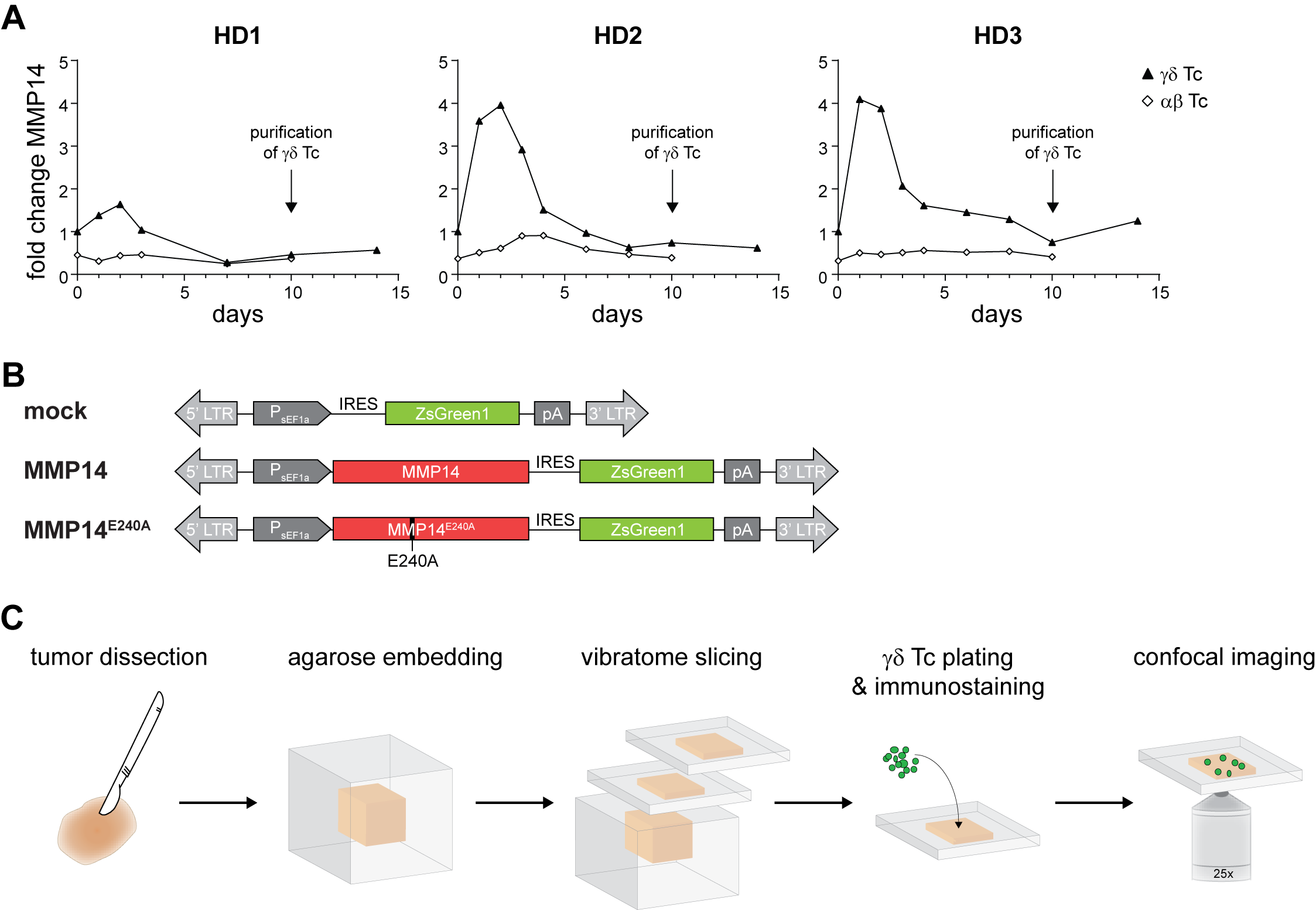


**Supplementary Fig. S3: MMP14 expression inγδ T cells.**

**(A)** Flow cytometry-based analysis of endogenous MMP14 expression in γδ (CD3^+^γδTCR^+^) and αβ (CD3^+^γδTCR^-^) T cells after the stimulation of PBMCs with ConA (10 µg/ml), IL-2 (10 ng/ml) and IL-4 (10 ng/ml). The MFI of MMP14 expression on γδ T cells on day 0 was set to 1 for normalization. αβ T cells were depleted via MACS from the cultures on day 10 after initial stimulation (arrow). Results for three healthy donors of γδ T cells are shown. **(B)** Schematic diagram of the lentiviral vectors used to express MMP14 or the catalytically inactive mutant MMP14^E240A^. **(C)** Experimental setup to analyze γδ T cell migration in viable slices of BCSC5 xenograft tumors. BCSC5 xenograft tumors were embedded in agarose and viable tissue slices of 350 μm thickness were cut using a vibratome. Slices were stained with fluorophore‑conjugated antibodies and CMFDA‑labeled γδ T cells were plated on top of the tumor slices. After allowing γδ T cells to infiltrate the tissue for 30 min, infiltrated cells were imaged by confocal microscopy. Modified from previously published protocols (27,44). CMFDA, 5-Chloromethylfluorescein diacetate; IRES, internal ribosomal entry site; LTR, long terminal repeat; pA, polyA; sEF1α, short elongation factor 1‑alpha.

**
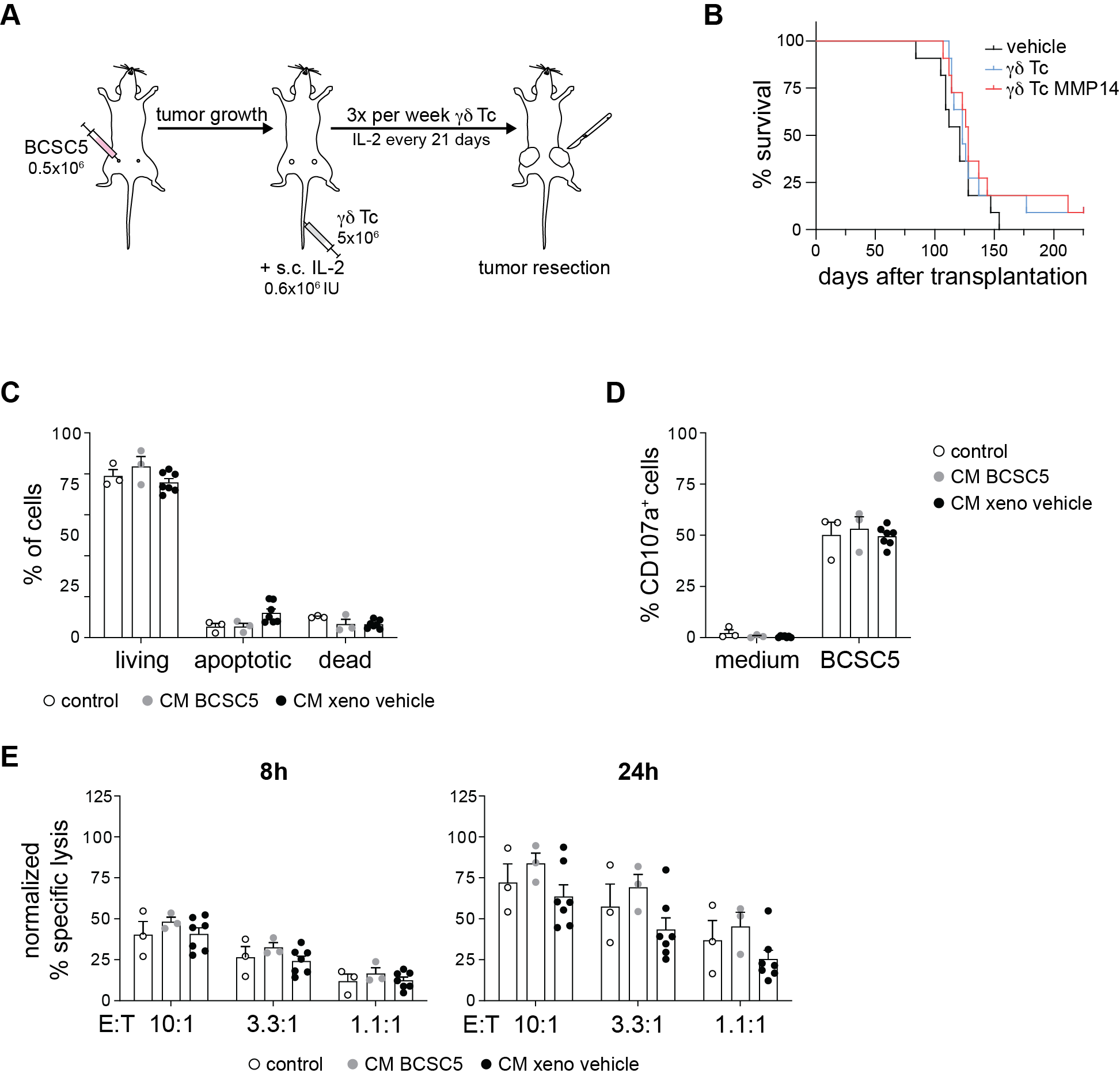
**

**Supplementary Fig. S4: γδ T cell therapy of BCSC5 xenografts in immunocompromised mice.**

**(A)** Schematic illustration of BCSC5 transplantation and γδ T cell immunotherapy in immunocompromised (NOD-SCID or Rag2^-/-^γc^-/-^**)** mice. 0.5x10^6^ BCSC5 cells were orthotopically transplanted into each fat pad of the two #4 mammary glands of 6-8 weeks old female mice. Treatment start was defined for each mouse individually when the first tumor reached a volume of at least 8 mm^3^. 5x10^6^γδ T cells were injected intravenously three times per week. In addition, mice received 0.6x10^6^ IU IL-2 (Proleukin S) on the day of treatment start and every 21 days until the end of the experiment. The end of the experiment was defined by a tumor volume of 800 mm^3^. **(B)** Kaplan-Meier plot of BCSC5 xenograft-bearing Rag2^-/-^γc^-/-^ mice upon treatment with γδ T cells (blue), γδ T cells expressing MMP14 (red) or vehicle control (black) (n=6-7 mice per group). Differences were not statistically significant, Log‑rank test (Mantel‑Cox). **(C)** Flow cytometry‑based assessment of γδ T cell viability after exposure to conditioned medium (CM) from BCSC5 culture cells or xenograft‑derived tumor cells (xeno). γδ T cells were cultured in CM for 24 h. CM was removed and cell viability was analyzed 24 h later. Living: Annexin V^-^/propidium iodide (PI)^-^, apoptotic: Annexin V^+^/PI^-^, dead: Annexin V^+^/PI^+^. Results for two healthy donors of γδ T cells from three independent experiments were pooled (means ± SEM). Two‑way ANOVA followed by Dunnet’s post hoc test comparing CM samples to respective controls. Differences were not statistically significant. **(D)** Flow cytometry-based analysis of degranulation by γδ T cells in response to BCSC contact for 3 h. γδ T cells were cultured in CM from BCSC5 culture cells or xenograft‑derived tumor cells for 24 h and then used for degranulation assays. The percentages of CD107a^+^ cells of γδTCR^+^‑gated cells for two healthy donors of γδ T cells from three independent experiments were pooled (means ± SEM). Two‑way ANOVA followed by Dunnet’s post hoc test comparing CM samples to respective controls. No significant differences were obtained. **(E)** *In vitro* killing of luciferase-expressing BCSC5 by γδ T cells after 8 h and 24 h at different effector to target (E:T) ratios. γδ T cells were cultured in CM from BCSC5 culture cells or xenograft‑derived tumor cells for 24 h and then used for cytotoxicity assays. Results for two healthy donors of γδ T cells from three independent experiments were pooled (means ± SEM). Two‑way ANOVA followed by Dunnet’s post hoc test comparing CM samples to respective controls. No significant differences were obtained.

**
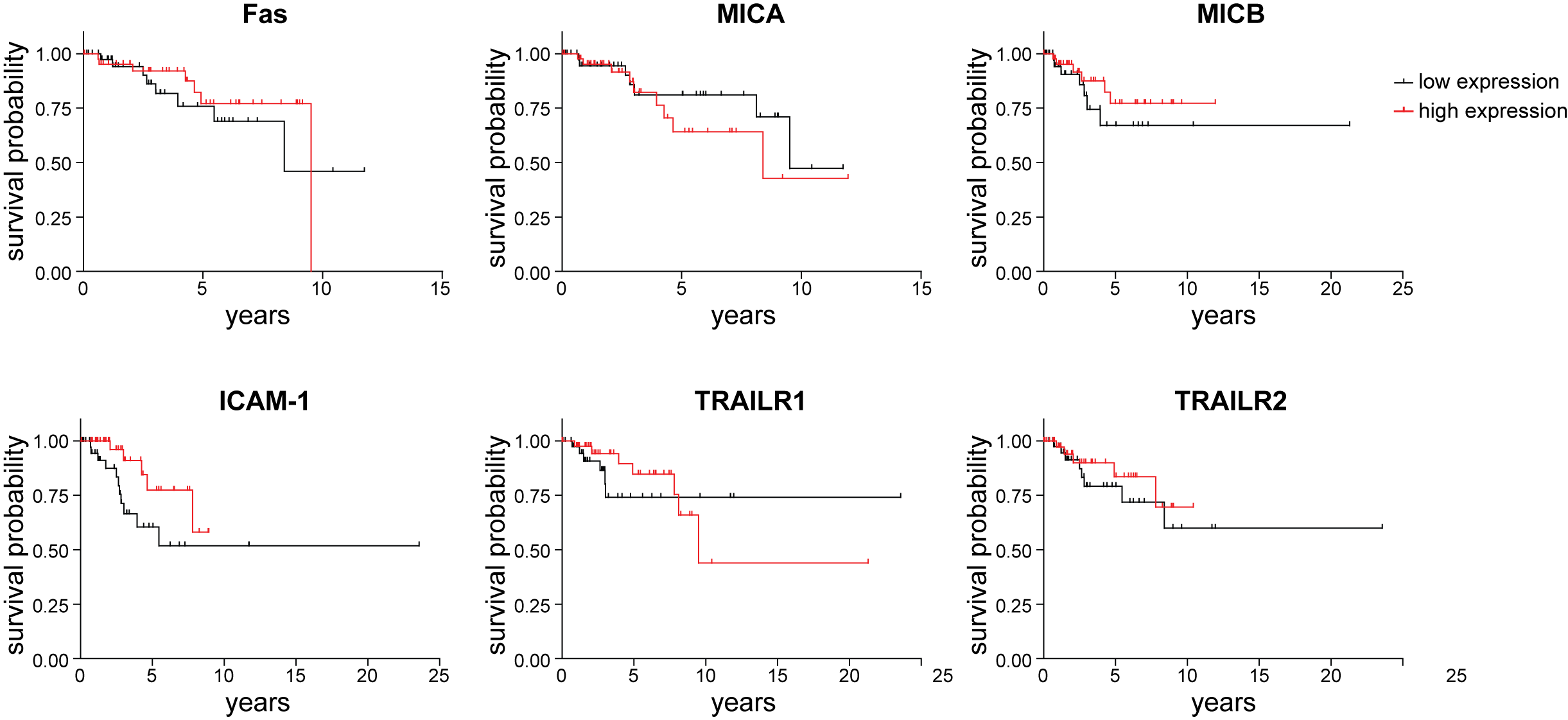
**

**Supplementary Fig. S5 (corresponds to Fig. 6): Killing of BCSC5 by γδ T cells requires multiple ligand-receptor interactions.** Cox regressions of progression-free survival for TNBC patients sorted by high (upper-quartile) and low (lower-quartile) expression of the indicated proteins. All individual proteins but MICA showed negative β cox coefficient indicating that patients with high expression exhibit a better survival prognosis. However, none of the individual cox p value was statistically significant (<0.05).


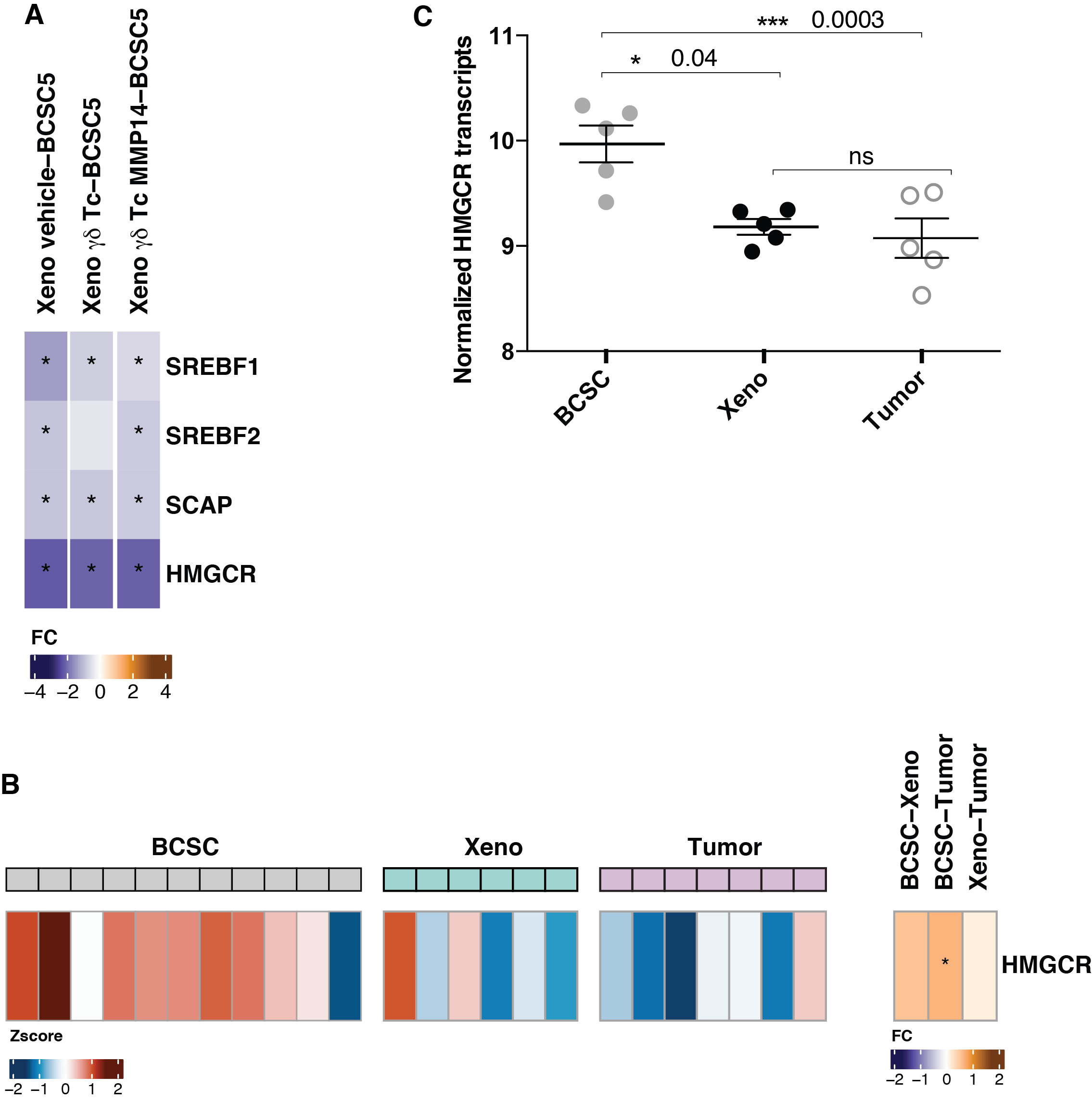


**Supplementary Fig. S6 (corresponds to Fig. 7): HMGCR is significantly downregulated in the xenograft-derived tumor cells and in the original patient tumors. (A)** Fc heat map showing up‑ and downregulated proteins in BCSC5 culture cells or xenograft‑derived tumors. Gold, positive log fold-change (FC) indicates higher expression in the first sample (Xeno vehicle, Xeno gd Tc or Xeno gd Tc-MMP14, as indicated) compared with BCSC5 culture cells; Purple, negative log FC. **(B)** Row wise Z‑score heatmap for HMGCR transcripts obtained from RNA microarray data. Samples are patient tumor-derived BCSC lines, BCSC xenograft-derived tumor cells and original patient tumors. Genes expressed significantly different are indicated with *. **(C)** The data of B were paired by patient, BCSC line and xenograft-derived tumors and analysed using one-way ANOVA followed by Tukey´s multiple comparison test. * p≤0.05, *** p≤0.001.

**
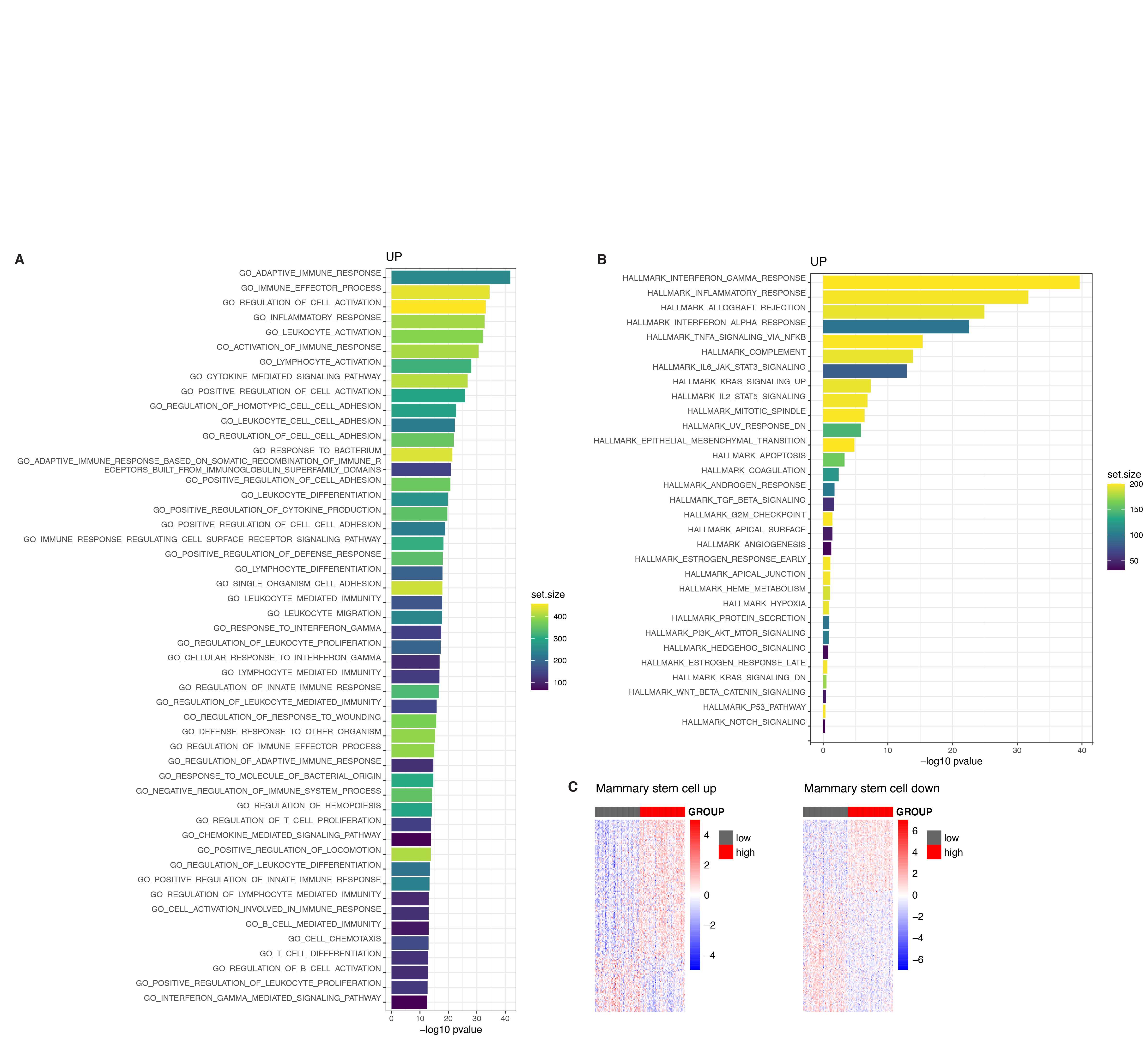
**

**Supplementary Fig. S7 (corresponds to Fig. 7): Gene enrichment analysis of TNBC patients.** TNBC patients were sorted by high (upper-quartile) and low (lower-quartile) average expression of BTN2A1, BTN3A1, Fas, MICB and ICAM-1 and a Generally Applicable Gene-set Enrichment (GAGE) analysis was performed using the **(A)** Hallmark and **(B)** GO databases. Color scale represents the number of annotated genes. **(C)** Row‑wise Z‑score heatmaps showing up‑ and downregulated mamary stemm cell signatures for TNBC patients clustered as in A.


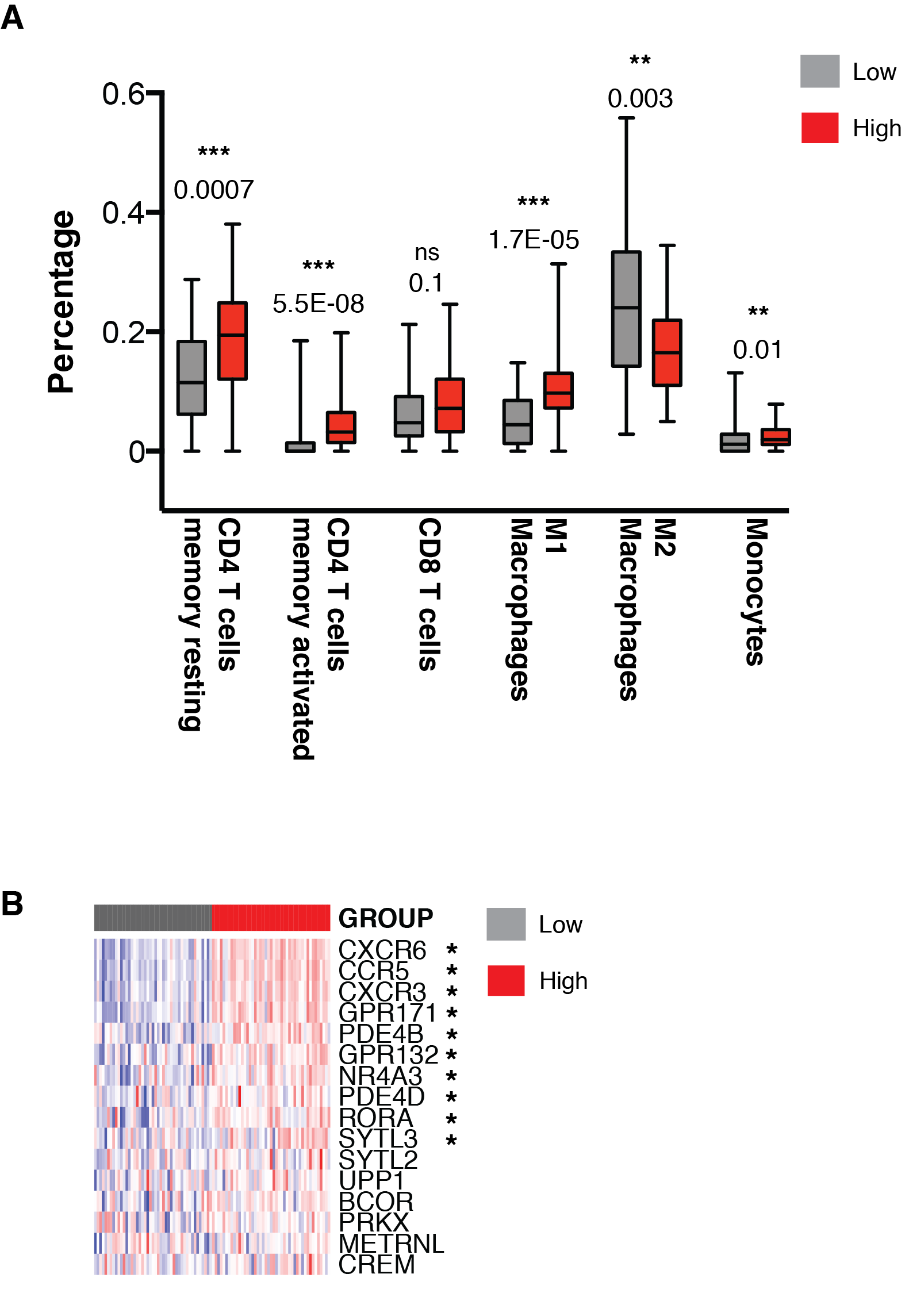


**Supplementary Fig. S8 (corresponds to Fig. 7): Immune profiling of TNBC patients. (A)** TNBC patients were sorted by high (upper-quartile) and low (lower-quartile) average clustered expression of BTN2A1, BTN3A1, Fas, MICB and ICAM-1 and the deep deconvolution CIBERSORT algorithm to deduce the immune cell composition. * p≤0.05, ** p≤0.01, *** p≤0.001. **(B)** Row wise Z‑score heatmap for a gene set specific for human γδ T cells according to (63). Out of 23 genes forming this set, only 16 were annotated in the database. Significantly different expressed genes are indicated with *.

**Supplementary Videos 1 and 2. MMP14 expression increases γδ T cell migration in BCSC5 tumor tissue.**

Migration of CMFDA‑labeled UT (Supplementary Video 1) or MMP14 expressing (Supplementary Video 2) γδ T cells (green) in vibratome sections of viable BCSC5 xenograft tumors. Shown are videos from BCSC5 xenograft tumor slices stained for EpCAM (blue) and fibronectin (red) to identify tumor cell regions and stromal compartments, respectively.
